# Diagnostic yield and clinical impact of germline sequencing in children with CNS and extracranial solid tumors—a nationwide, prospective Swedish study

**DOI:** 10.1016/j.lanepe.2024.100881

**Published:** 2024-03-19

**Authors:** Bianca Tesi, Kristina Lagerstedt Robinson, Frida Abel, Teresita Díaz de Ståhl, Sara Orrsjö, Anna Poluha, Maria Hellberg, Sandra Wessman, Sofie Samuelsson, Tony Frisk, Hartmut Vogt, Karin Henning, Magnus Sabel, Torben Ek, Niklas Pal, Per Nyman, Geraldine Giraud, Joakim Wille, Cornelis Jan Pronk, Ulrika Norén-Nyström, Magnus Borssén, Maria Fili, Gustav Stålhammar, Nikolas Herold, Giorgio Tettamanti, Carolina Maya-Gonzalez, Linda Arvidsson, Anna Rosén, Katja Ekholm, Ekaterina Kuchinskaya, Anna-Lotta Hallbeck, Margareta Nordling, Pia Palmebäck, Per Kogner, Gunilla Kanter Smoler, Päivi Lähteenmäki, Susanne Fransson, Tommy Martinsson, Alia Shamik, Fredrik Mertens, Richard Rosenquist, Valtteri Wirta, Emma Tham, Pernilla Grillner, Johanna Sandgren, Gustaf Ljungman, David Gisselsson, Fulya Taylan, Ann Nordgren

**Affiliations:** a Department of Molecular Medicine and Surgery, Karolinska Institutet, Stockholm, Sweden; b Clinical Genetics and Genomics, Karolinska University Hospital, Solna, Sweden; c Department of Medicine, Center for Hematology and Regenerative Medicine, Karolinska Institutet, Stockholm, Sweden; d Department of Clinical Genetics and Genomics, Sahlgrenska University Hospital, Gothenburg, Sweden; e Department of Laboratory Medicine, Institute of Biomedicine, Sahlgrenska Academy, University of Gothenburg, Gothenburg, Sweden; f Department of Oncology-Pathology, Karolinska Institutet, Stockholm, Sweden; g Clinical Pathology and Cancer Diagnostics, Karolinska University Hospital, Stockholm, Sweden; h Clinical Genetics, Uppsala University Hospital, Uppsala, Sweden; i Department of Immunology, Genetics and Pathology, Uppsala University, Uppsala, Sweden; j Department of Clinical Genetics, Pathology and Molecular Diagnostics, Office of Medical Services, Region Skåne, Lund, Sweden; k Department of Pediatric Hematology and Oncology, Karolinska University Hospital, Stockholm, Sweden; l Childhood Cancer Research Unit, Department of Women’s and Children’s Health, Karolinska Institutet, Stockholm, Sweden; m Crown Princess Victoria Children’s Hospital, and Division of Children’s and Women’s Health, Department of Biomedical and Clinical Sciences, Linköping University, Linköping, Sweden; n Department of Pediatrics, Institute of Clinical Sciences, Sahlgrenska Academy, University of Gothenburg, Sweden; o Queen Silvia Children’s Hospital, Sahlgrenska University Hospital, Gothenburg, Sweden; p Department of Health, Medicine and Caring Sciences, Linköping University, Linköping, Sweden; q Centre for Medical Image Science and Visualization (CMIV), Linköping University, Linköping, Sweden; r Department of Immunology, Genetics, and Pathology, Science for Life Laboratory, Rudbeck Laboratory, Uppsala University, Uppsala, Sweden; s Pediatric Oncology, Uppsala University Children’s Hospital, Uppsala, Sweden; t Department of Women’s and Children’s Health, Uppsala University, Sweden; u Childhood Cancer Center, Skåne University Hospital, Lund, Sweden; v Division of Molecular Hematology/Wallenberg Center for Molecular Medicine, Lund University, Sweden; w Department of Clinical Sciences, Pediatrics, Umeå University, Umeå, Sweden; x Division of Eye and Vision, Department of Clinical Neuroscience, Karolinska Institutet, Stockholm, Sweden; y St. Erik Eye Hospital, Stockholm, Sweden; z Unit of Epidemiology, Institute of Environmental Medicine, Karolinska Institutet, Stockholm, Sweden; aa Department of Radiation Sciences, Oncology, Umeå University, Umeå, Sweden; ab Department of Clinical Genetics, Linköping University Hospital, Linköping, Sweden; ac Department of Biomedical and Clinical Sciences, Linköping University, Linköping, Sweden; ad Genomic Medicine Center Karolinska, Karolinska University Hospital, Stockholm, Sweden; ae Science for Life Laboratory, Department of Microbiology, Tumour and Cell Biology, Karolinska Institutet, Stockholm, Sweden; af Science for Life Laboratory, School of Engineering Sciences in Chemistry, Biotechnology and Health, KTH Royal Institutet of Technology, Stockholm, Sweden

**Keywords:** Childhood cancer predisposition, Whole-genome sequencing, Germline variants, Somatic mutations

## Abstract

**Background:**

Childhood cancer predisposition (ChiCaP) syndromes are increasingly recognized as contributing factors to childhood cancer development. Yet, due to variable availability of germline testing, many children with ChiCaP might go undetected today. We report results from the nationwide and prospective ChiCaP study that investigated diagnostic yield and clinical impact of integrating germline whole-genome sequencing (gWGS) with tumor sequencing and systematic phenotyping in children with solid tumors.

**Methods:**

gWGS was performed in 309 children at diagnosis of CNS (n = 123, 40%) or extracranial (n = 186, 60%) solid tumors and analyzed for disease-causing variants in 189 known cancer predisposing genes. Tumor sequencing data were available for 74% (227/309) of patients. In addition, a standardized clinical assessment for underlying predisposition was performed in 95% (293/309) of patients.

**Findings:**

The prevalence of ChiCaP diagnoses was 11% (35/309), of which 69% (24/35) were unknown at inclusion (diagnostic yield 8%, 24/298). A second-hit and/or relevant mutational signature was observed in 19/21 (90%) tumors with informative data. ChiCaP diagnoses were more prevalent among patients with retinoblastomas (50%, 6/12) and high-grade astrocytomas (37%, 6/16), and in those with non-cancer related features (23%, 20/88), and ≥2 positive ChiCaP criteria (28%, 22/79). ChiCaP diagnoses were autosomal dominant in 80% (28/35) of patients, yet confirmed *de novo* in 64% (18/28). The 35 ChiCaP findings resulted in tailored surveillance (86%, 30/35) and treatment recommendations (31%, 11/35).

**Interpretation:**

Overall, our results demonstrate that systematic phenotyping, combined with genomics-based diagnostics of ChiCaP in children with solid tumors is feasible in large-scale clinical practice and critically guides personalized care in a sizable proportion of patients.

**Funding:**

The study was supported by the Swedish Childhood Cancer Fund and the 10.13039/501100005348Ministry of Health and Social Affairs.

**Research in context**

**Evidence before this study**

Genomic studies with a genotype-first approach conducted across childhood cancers have identified germline variants in genes predisposing to childhood cancer in 5%–18% of all children with cancer. The reported diagnostic yields are influenced by factors such as differences in study cohorts, study design, and the definition of a positive germline finding. Studies based on phenotype-first approach argue for the use of screening tools to select children with possible childhood cancer predisposition for further germline testing. For either approach, most studies have been performed in cohorts of children with high-risk or relapsed disease.

**Added value of this study**

In this study, conducted within a Swedish national infrastructure for precision medicine, we combine germline and tumor whole genome sequencing with systematic phenotyping on a nationwide and prospective cohort of 309 children included at diagnosis of any solid malignancy between 1st May 2021 and 31st December 2022. Using an in-silico panel of 189 genes, we uncover an overall prevalence of known childhood cancer predisposition syndromes of 11% and a diagnostic yield of 8% for childhood cancer predisposition syndromes not known at cancer diagnosis. We show how systematic phenotyping stratifies patients into different pre-test probabilities for germline findings and the power of paired somatic analysis for interpretation of germline findings. Using retinoblastoma as example, we demonstrate how integrated germline and tumor analyses can also be used to refine surveillance recommendations for siblings.

**Implications of all the available evidence**

Genomics-based diagnostics of ChiCaP in children with solid tumors, combined with systematic clinical phenotyping, is feasible in large-scale clinical practice and critically guides personalized care in a sizable proportion of patients. Systematic phenotyping is important for genotype–phenotype correlations and to identify patients with high suspicion of ChiCaP syndrome or other genetic disorders for which extended germline testing is warranted.

## Introduction

Childhood cancer encompasses a heterogeneous group of rare malignancies. About 1 in 300 children develop cancer by the age of 18 which translates to about 340 children diagnosed with a malignancy every year in Sweden.^1^ Childhood cancer predisposition (ChiCaP) syndromes are increasingly recognized as contributing factors to childhood cancer development.^2^ Hence, germline testing is becoming a key part of integrative genomics efforts for improved care of children with cancer.^3^, ^4^, ^5^, ^6^ Indeed, the recognition of a ChiCaP syndrome in a child diagnosed with cancer may inform clinical management in terms of treatment adjustment, surveillance, and identification of family members at risk. Genomic studies with a genotype-first approach conducted across childhood cancers have identified germline predisposition in 5%–18% of all children with cancer.^7^, ^8^, ^9^, ^10^ Differences in study cohorts, study design, and definition of a positive germline finding are all factors that influence diagnostic yields. A recent retrospective study of a phenotype-first approach for ChiCaP diagnostics demonstrated a diagnostic yield of 8.6%.^11^ However, phenotype-first approaches require robust clinical screening tools to identify patients with ChiCaP syndromes.^12^

Since 2017, the Genomic Medicine Sweden (GMS) initiative is building a national infrastructure for implementation of precision medicine based on offering whole-genome sequencing (WGS) within the healthcare system to patients with cancer and rare diseases.^13^, ^14^, ^15^ As part of the GMS initiative and with the goal of implementing integrative genomics in Swedish childhood cancer care, germline and tumor WGS have been offered prospectively since 2021 to all children diagnosed with CNS and extracranial solid tumors.^16^

Here we present the results from the first 309 consecutive children with CNS or extracranial solid tumors included in the GMS Childhood Cancer Predisposition (GMS-ChiCaP) study. Thanks to a population-based cohort, a stringent definition of a pathogenic germline finding, integration with tumor molecular data and systematic assessment of clinical indicators of germline predisposition using a dedicated form our findings can help shape worldwide clinical routines to diagnose childhood cancer predisposition.

## Methods

### Study design, patients, and workflow

**Fig. 1:**
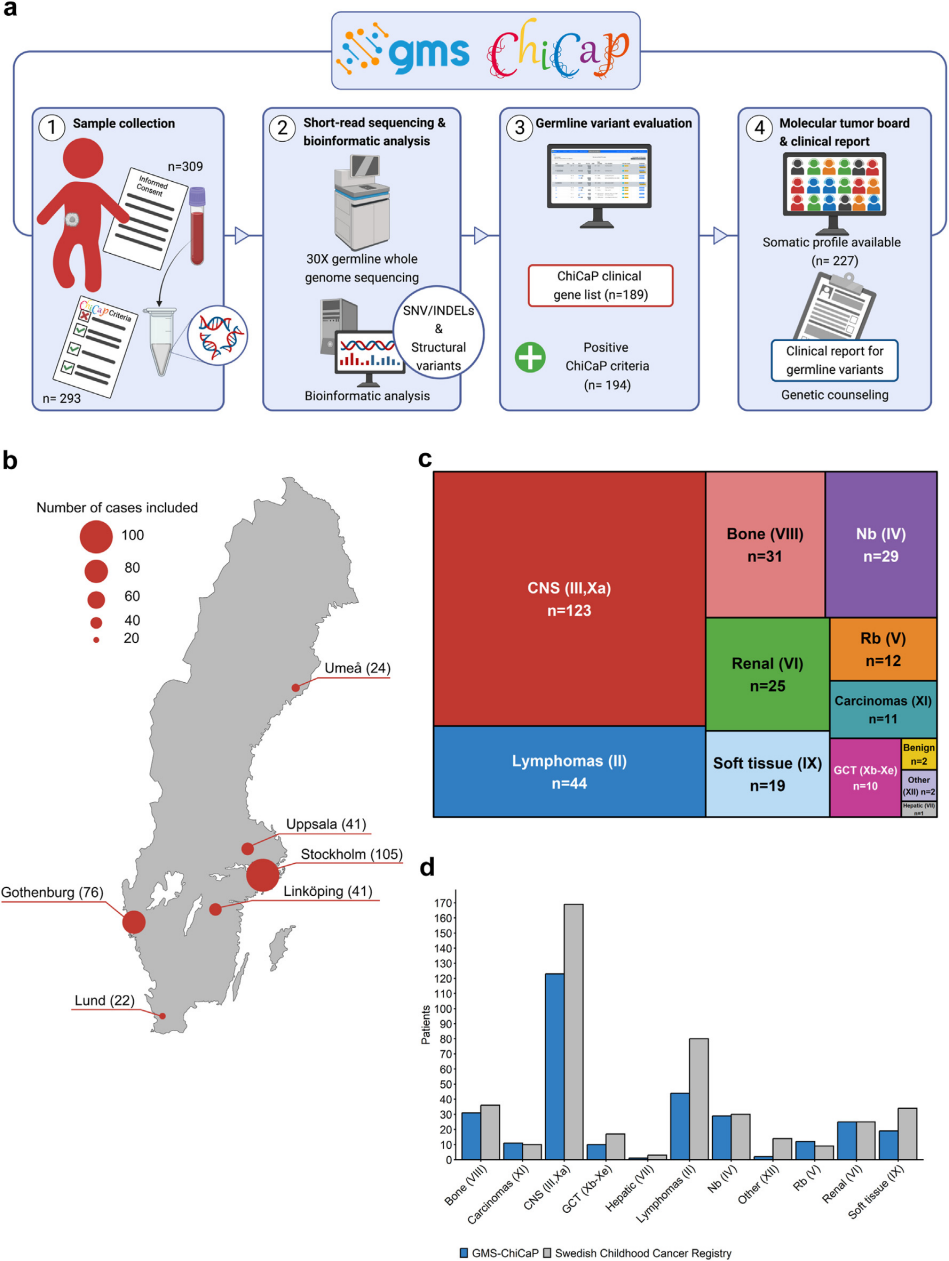
**Overview of study design and study cohort. a)** Study design, figure created with BioRender.com; **b)** Nationwide inclusion. The Stockholm node includes both the Pediatric Oncology Department of Karolinska University Hospital and the Ocular Oncology Department of St. Erik Eye Hospital. Number of patients included by each center are presented in parenthesis (n). **c)** Treemap of the different tumors included. Diagnoses were classified according to International Classification of Childhood Cancer Third edition (ICCC-3). ICCC-3 groups are presented in parenthesis. CNS tumors emcompass both group III (n = 121) and group Xa (n = 2). Lymphoma (II) group includes also reticuloendothelial neoplasms. Nb = neuroblastoma; Rb = retinoblastoma; GCT = germ cell tumor. **d)** Comparison between study cohort (GMS-ChiCaP) and tumor diagnoses made in Sweden during the study period according to data from the Swedish Childhood Cancer Registry, grouped according to ICCC-3. Nb = neuroblastoma; Rb = retinoblastoma; GCT = germ cell tumor. CNS tumors emcompass both group III and group Xa.

An overview of the study design is given in Fig. 1a. Patients <18 years of age diagnosed between 1st May 2021 and 31st December 2022 with a primary CNS or extracranial solid malignancy at one of the six pediatric oncology centers in Sweden or at the St Erik Eye Hospital in Stockholm were offered to participate in the study (Fig. 1b). All patients were eligible for inclusion as long as written consent was given and a blood sample for extraction of germline DNA collected. In addition, parental blood samples were collected. Patients with a known clinical or molecularly defined ChiCaP syndrome at diagnosis were not excluded. Tumor analysis required availability of biopsy material and at least 40% tumor cell content as assessed by microscopy.^16^ At inclusion, a standardized assessment for underlying ChiCaP was performed with the help of a form built upon existing clinical screening tools (Supplementary methods), hereafter referred to as the “ChiCaP criteria form”. Collected information was summarized into four main ChiCaP criteria: “family history”: family history of cancer or parental consanguinity; “multiple primary” if the child had multiple primary malignancies (synchronous or metachronous); “other features”, if the child had non-cancer related features; “malignancy type”, if the child had a tumor type with stronger association to predisposition based on previous knowledge (Supplementary methods). While patient recruitment occurred at seven centers, genetic analyses, adjusted to local routines, were centralized to three nodes (Supplementary methods).^16^ The individual patients’ results were presented and discussed at multidisciplinary molecular tumor boards before they were reported in written form to the treating physician and included in patients’ medical charts. Follow-up information was collected from medical charts. Tumor types are described according to WHO 2021 classification of central nervous system tumors and pediatric tumors,^17^^,^^18^ with the exception for a few new entities, and grouped according to the third edition of International Classification of Childhood Cancer (ICCC-3).^19^ CNS tumors were further grouped into categories.^20^ Extracranial solid tumors were defined as all solid tumors not belonging to ICCC-3 group III (CNS and miscellaneous intracranial and intraspinal neoplasms) or group Xa (intracranial and intraspinal germ cell tumors).

### Germline WGS and data analysis

Genomic DNA was extracted from peripheral blood according to standard procedures. Germline WGS (gWGS; 30x coverage) was performed leveraging established bioinformatic pipelines in clinical use for diagnostics of rare diseases at the three analysis nodes (Supplementary methods). Depending on local pipelines, human genome assembly hg19 or hg38 was used. gWGS data were filtered for variants affecting 189 known cancer predisposing genes (Supplementary methods and Table S1). Results from a 50-gene list were previously published for 78 children included in this study.^16^ Germline single nucleotide variants (SNVs), small insertions and deletions (INDELs), and structural variants (SVs) were interpreted and classified by a clinical laboratory geneticist and a clinical geneticist. Variants classified as pathogenic (P) or likely pathogenic (LP) according to American College of Medical Genetics and Genomics (ACMG) guidelines or gene-specific guidelines when applicable^21^^,^^22^ following the syndrome’s known inheritance pattern were reported to the treating physicians and are presented here. Heterozygous P/LP variants in genes associated with autosomal recessive ChiCaP syndromes were not reported unless affecting genes also associated with autosomal dominant highly penetrant adult onset cancer predisposition syndromes (Supplementary methods). In selected patients, segregation analysis with parental samples was performed—e.g., to prove *de novo* origin of the variant—prior to variant classification as P/LP. Depending on local laboratory routines, some variants were verified with a secondary method before reporting.

### Tumor sequencing and data analysis

DNA derived from fresh frozen tumor was analyzed by WGS (90x coverage) (Supplementary methods). DNA derived from formalin-fixed paraffin-embedded (FFPE) tumor was analyzed by whole exome sequencing for five retinoblastomas for which fresh frozen material was not available. Second-hit analysis was performed on tumor data from patients with P/LP germline variants in tumor suppressor genes. Second-hits were broadly classified as: 1) second mutation: somatic SNVs/INDELS affecting the same gene; 2) copy neutral loss of heterozygosity (CN-LOH) defined as CN-LOH detected by analysis of B-allele frequency or variant allele frequency >70% for the germline variant in absence of a copy number change; 3) structural variant. An SNV-based tumor mutation burden (TMB) was calculated by dividing the number of high-confidence (coverage > 10 i both tumor/normal; allele depth in tumor >3, allele fraction > 0.05) somatic SNVs identified in tumor WGS after tumor-normal analyses by the size of the reference genome used for assembly (hg19).^6^ Single Base Substitutions (SBS) mutational signatures were analyzed using AlexandrovLab R packages in selected patients.^23^ Assignment of known COSMIC mutational signatures v3.3 to individual samples was performed with the *cosmic_fit* function from SigProfilerAssignment using vcf somatic mutation calling files derived from the TNscope variant caller tool (Sentieon) as input.

Analysis of somatic *RB1* mutations was performed in all patients of retinoblastoma without germline *RB1* variants. To this analysis we also included children diagnosed with retinoblastoma after 31st December 2022 and before 30th July 2023 (n = 4). These four children are excluded for all calculations pertaining the cohort of 309 children.

### Ethics and data availability

The study is approved by the Swedish Ethical Review Authority (permit no. 2020-03827, 2021-05916-02, and 2022-07167-02). Written informed consent was obtained for all study subjects included from the children’s parents or from legal guardians. For patients of age ≥ 15 years direct consent was also obtained. Information about the study was given by a trained pediatrician, pediatric oncologist, oncologist, or ocular oncologist. The signed informed consent included the information about return of germline findings consistent with ChiCaP syndromes, as well as other potential actionable incidental findings, if made. Raw and analyzed genomic data from the study have been deposited at the Swedish Childhood Tumor Biobank and are available for secondary use within research projects on pediatric cancer through a controlled data access procedure. This procedure is also described in the study consent signed by all study subjects and/or parents or legal guardians.

### Statistical analyses

We used logistic regression models to evaluate the association between ChiCaP diagnosis and the clinical information collected through the ChiCaP criteria form, presented as odds ratio (OR) with 95% confidence intervals (CI). Analyses were adjusted for age at cancer diagnosis, sex, and tumor type. We used the binomial distribution to report confidence intervals for discussing prevalence and diagnostic yields of ChiCaP syndromes. All analyses were performed using R Statistical Software (v4.2.3; R Core Team 2023).

### Role of the funding source

The funders of the study described above were not involved during study design, data collection, data analysis, data interpretation, or writing of the report.

## Results

### Baseline characteristics

Between May 2021 and December 2022, gWGS was performed in 309 children (median age at diagnosis, 7 [range: 0–17] years; 140 girls [45%]) recruited nationwide at diagnosis of a CNS (40%, 123/309) or an extracranial solid tumor (60%, 186/309) (Fig. 1b and c, Supplementary Fig. S1a, Table 1, Supplementary Table S2). The most common types of extracranial solid tumors were lymphomas and reticuloendothelial neoplasms (n = 44), malignant bone tumors (n = 31), neuroblastomas (n = 29), and renal tumors (n = 25) (Fig. 1c). Among CNS tumors, the most common were low-grade astrocytomas (n = 64), followed by medulloblastomas (n = 19), and high-grade astrocytomas (n = 16) (Supplementary Fig. S1b). To assess the population coverage of the GMS-ChiCaP study we conducted a comparative analysis using data from the Swedish Childhood Cancer Registry (SCCR), a National Quality Registry with at least 93% completeness for cancer diagnoses in children <15 years of age.^1^ Compared to data obtained from the Swedish Childhood Cancer Registry for the study period, our cohort was well representative for all major malignancy groups, yet with lower coverage for lymphomas and CNS tumors (Fig. 1d, Supplementary methods). Findings were discussed at local multidisciplinary rounds attended by clinical and laboratory geneticists, pathologists, pediatric oncologists, and participating researchers for a total of 74 multidisciplinary rounds carried out during the study time.

**Table 1:** Patient characteristics.

Characteristic	N	Overall, N = 309^a^	ChiCaP diagnosis		OR	95% CI
			No, N = 274^a^	Yes, N = 35^a^		
Age at diagnosis	309	7.0 (3.0, 13.0)	7.0 (3.0, 13.0)	7.0 (2.5, 12.0)		
Sex	309					
Female		140 (45%)	125 (46%)	15 (43%)		
Male		169 (55%)	149 (54%)	20 (57%)		
Tumor type	309					
CNS tumor		123 (40%)	109 (40%)	14 (40%)		
Extracranial solid tumor		186 (60%)	165 (60%)	21 (60%)		
≥1 positive ChiCaP criteria	293	194 (66%)	161 (62%)	33 (94%)	10.2	3.0–64.1
Family history for cancer	290	64 (22%)	51 (20%)	13 (38%)	2.5	1.2–5.4
Malignancy type	309	131 (42%)	107 (39%)	24 (69%)	3.8	1.8–8.8
Multiple primary tumors	291	10 (3.4%)	5 (2.0%)	5 (14%)	9.2	2.4–35.9
≥1 additional features	293	88 (30%)	68 (26%)	20 (57%)	3.9	1.9–8.4
≥2 additional features	293	26 (8.9%)	16 (6.2%)	10 (29%)	6.4	2.5–16.0
ID or DD	293	11 (3.8%)	6 (2.3%)	5 (14%)	7.2	2.0–25.7

N = number of valid cases; ID = intellectual disability; DD = developmental delay; OR = odds ratio; CI = confidence intervals.

Logistic regression model adjusted for age at diagnosis, sex, and tumor type.

a Median (IQR); n (%).

### Frequency and spectrum of germline variants

**Fig. 2:**
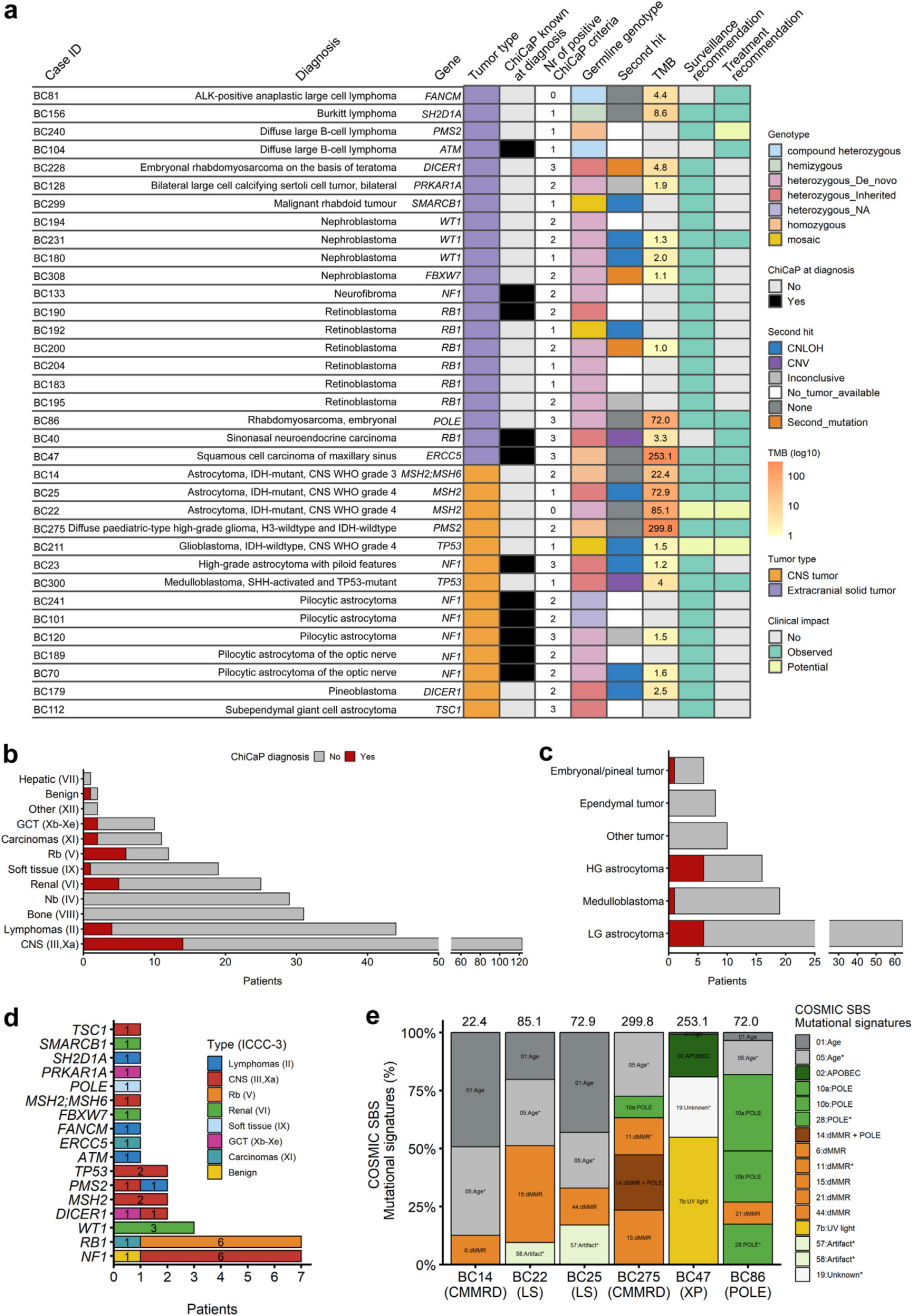
**Germline findings. a)** Tile plot where each row represents a unique patient with germline P/LP findings. The row names indicate the patient ID, the tumor type and the mutated gene. The columns present, from left to right, [column title, values], “Case ID”; “Diagnosis” WHO 2021 tumor type; “Gene” mutated in germline; “Tumor type”, CNS or extracranial solid tumor; “ChiCaP known at diagnosis”, if the ChiCaP syndrome was known at study inclusion (‘Yes’) or discovered within the study (‘No’); “Nr of positive ChiCaP criteria”, the number of fulfilled ChiCaP criteria (range 0–4); “Germline genotype”, the genotype of the germline finding with‘heterozygous_NA’ for patients where parental samples were not available; “Second hit”, presence and type of second hit in the same gene (‘inconclusive’ refers to tumor data were no somatic findings were made, possibly suggesting a non-representative sample); ‘CNLOH’ = copy number neutral loss of heterozygosity; ‘CNV’ = copy number variant); “TMB”, log10 tumor mutation burden, “Surveillance recommendation”, whether the ChiCaP finding resulted in potential or observed ChiCaP-informed follow-up; “Treatment recommendation”, whether the ChiCaP finding results in potential or observed ChiCaP-informed treatment choices. **b)** Number of patients diagnosed with ChiCaP in different tumor types according to main ICCC-3 groups. CNS tumors emcompass both group III (n = 121) and group Xa (n = 2). Nb = neuroblastoma; Rb = retinoblastoma; GCT = germ cell tumor. **c)** Number of patients with ChiCaP syndromes in categories of CNS tumors. HG = high-grade; LG = low-grade. **d)** Histogram of mutated genes in relation to main ICCC-3 groups. **e)** Proportion of somatic single base substitution (SBS) COSMIC mutational signatures detected by WGS in the tumor of the six patients with TMB > 10. BC14 with germline homozygous variants in *MSH2* and *MSH6* genes (CMMRD) and astrocytoma, IDH-mutant, CNS WHO grade 3; BC22 with germline heterozygous variant in *MSH2* (Lynch syndrome, LS) and astrocytoma IDH-mutant CNS WHO grade 4; BC25 with germline heterozygous variant in *MSH2* (LS) and astrocytoma IDH-mutant CNS WHO grade 4; BC275 with germline homozygous variants in *PMS2* (CMMRD) and diffuse pediatric-type high-grade glioma, H3-wildtype and IDH-wildtype; BC47 with germline homozygous variant in *ERCC5* (Xeroderma pigmentosum, XP) and BC86 with germline heterozygous variant in *POLE* and embryonal rhabdomyosarcoma. Signatures labeled with “∗” have unclear evidence for real signature according to COSMIC and are labeled according to their proposed etiology in the COSMIC database. SBS11 has been associated also with temozolomide chemotherapy, but BC275 was treatment naive at sampling. Different signatures with same etiology, either validated or proposed (∗) where assigned the same color. The calculated TMB for the specific tumor is displayed on top of each bar.

gWGS data for the 309 children included were analyzed for P/LP SNVs/INDELs and SVs in 189 genes associated with childhood cancer predisposition. Overall, 38 variants affecting 17 genes in 35 children were identified as P/LP and reported to the treating physician and participating families (Supplementary Table S3, Fig. 2a). SNVs (n = 22) or INDELs (n = 10) constituted 84% (32/38) of all reported variants, while 16% (6/38) represented deletions, of which three were intragenic and three microdeletions. Across malignancy types, this translates to an overall prevalence of 11% (35/309) for well-established ChiCaP syndromes in our cohort. The prevalence of ChiCaP was similar for CNS tumors (11.4%, 14/123) and extracranial solid tumors (11.3%, 21/186), but higher in specific tumor subgroups such as retinoblastomas (50%, 6/12), high-grade astrocytomas (37%, 6/16), and renal tumors (20%, 5/25) (Fig. 2b and c). ChiCaP syndromes occurred in 9% (4/44) of children diagnosed with lymphomas, while none was found among children diagnosed with neuroblastoma or malignant bone tumors (Fig. 2b). Overall, 69% (24/35) of ChiCaP diagnoses were unknown at study inclusion (Fig. 2a), thus 8% (24/298) with no known ChiCaP diagnosis at inclusion received a molecular diagnosis from the study (Supplementary Fig. S2a–c). All the eleven patients with known ChiCaP diagnoses had well-established ChiCaP syndromes, either easily recognizable clinically (n = 9) or with strong association with a specific tumor type (n = 2) (Fig. 2a).

Thanks to stringent definition of a pathogenic germline finding, all genotypes were consistent with the known inheritance model for the respective ChiCaP syndrome (Fig. 2a), being autosomal dominant in 80% (28/35), autosomal recessive in 17% (6/35), and X-linked in 3% (1/35). Parental blood samples were available for 90% (63/70) of parents to children with ChicaP findings. Out of the 28 autosomal dominant diagnoses, d*e novo* origin was confirmed in 18 (64%, 18/28) patients, of which three were mosaic in blood, while eight patients inherited the P/LP variant from a parent. Parental origin could not be assessed in two children with Neurofibromatosis type 1 due to unavailable parental samples, but neither had family history.

The germline genetic landscape was heterogeneous with 17 mutated genes and differed between CNS and extracranial solid tumors (Fig. 2d, Supplementary Table S3). Among children with CNS tumors, the most frequently mutated genes were *NF1* (n = 6), mismatch repair (MMR) genes (n = 4), and *TP53* (n = 2). All six children with LP/P variants in the *NF1* gene had a known Neurofibromatosis type 1 diagnosis; five were diagnosed with pilocytic astrocytoma of which two affecting the optic pathway; one with a high-grade astrocytoma with piloid features (Fig. 2a, Supplementary Table S3). All four children with LP/P variants in MMR genes had high-grade astrocytomas. Two children, with *IDH1*-mutant high-grade astrocytomas and germline heterozygous *MSH2* variants, were diagnosed with primary MMR-deficient *IDH1*-mutant astrocytomas^24^ on the basis of Lynch syndrome, while the other two had bi-allelic MMR variants and were diagnosed with constitutional mismatch repair deficiency (CMMRD) syndrome. Among extracranial solid tumors, the ChiCaP diagnoses were more heterogeneous and included six children with retinoblastoma and pathogenic variants in *RB1*, three children with Wilms tumor and pathogenic variants in *WT1*, and one child each with pathogenic variants in *DICER1, TP53, NF1, PMS2, ATM, ERCC5, FANCM, FBXW7, POLE, PRKAR1A, SH2D1A,* and *SMARCB1* (Fig. 2a and d).

### Somatic variant analysis supports germline variant interpretation

Somatic data were available from 69% (24/35) children with germline findings (Supplementary Table S4). Of these, 21 had complementary tumor WGS data enabling a paired analysis, 2 had complementary tumor WES data (from FFPE material), and 1 patient had available tumor SNP array data performed as part of routine diagnostics. Second-hit analysis was performed in 19 children with an autosomal dominant ChiCaP syndrome, revealing a genetic second hit in 73% (14/19) (Fig. 2a, Supplementary Table S4). The analysis was inconclusive for three tumors most likely due to low fraction of tumor cells in the analyzed tumor tissue. In the remaining two patients–BC22 (astrocytoma, IDH-mutant, CNS WHO grade 4 with a germline heterozygous *MSH2* variant) and BC86 (embryonal rhabdomyosarcoma and prior glioblastoma with a germline *POLE* variant)–with no apparent genetic second hit, we found high TMB and COSMIC mutational signatures consistent with defective MMR and defective polymerase epsilon exonuclease proofreading, respectively (Fig. 2a and e). Despite lack of a somatic second hit in *MSH2*, BC22 was interpreted as having Lynch syndrome rather than CMMRD due to lack of clinical signs of CMMRD and the preserved expression of MSH2 and MSH6 in normal cells based on immunohistochemistry. High TMB and mutational signature of MMR deficiency was also observed in two additional patients with CMMRD (BC14 and BC275) and one more patient with Lynch syndrome (BC25) (Fig. 2e). In patient BC47 with Xeroderma pigmentosum (XP) and a squamous cell carcinoma of maxillary sinus a high TMB and a somatic mutational signature consistent with UV-caused mutagenesis was observed (Fig. 2e). Therefore, a concordant somatic mutation profile—i.e., second hit or mutational signature–was observed in 90% (19/21) of children with ChiCaP syndromes and informative tumor data, which in turn corroborates the pathogenicity of the reported germline findings.

### Clinical indicators of childhood cancer predisposition

**Fig. 3:**
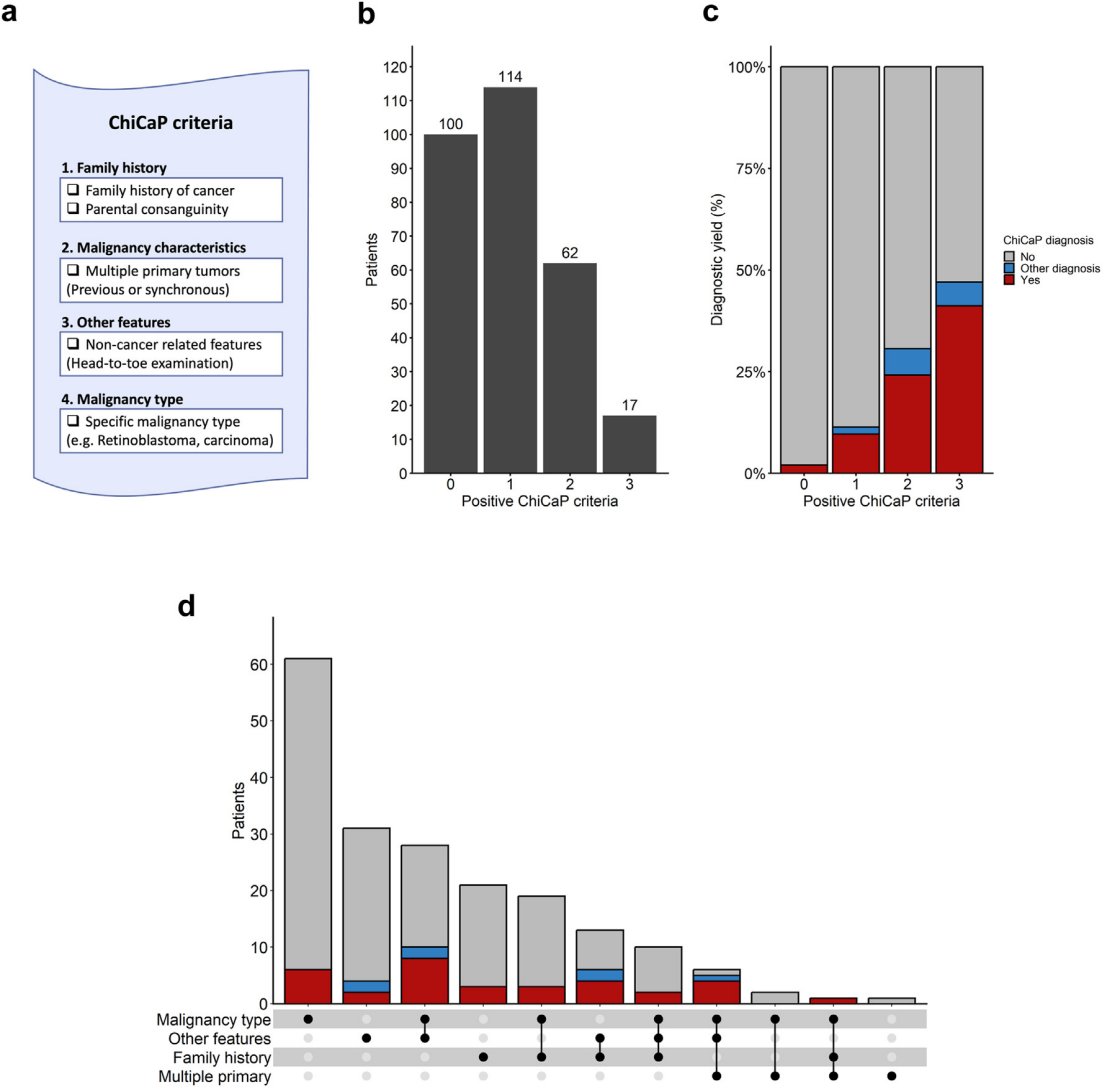
**ChiCaP indicators. a)** Structure of ChiCaP criteria form. The ChiCaP criteria form is described in details in Supplementary methods. **b)** Number of patients fulfilling 0–3 ChiCaP criteria. No patient fulfilled 4 ChiCaP criteria. **c)** Diagnostic yield according to the number of fulfilled ChiCaP criteria. A and B include the 293 patients with ChiCaP criteria information available. “Other diagnosis” refers to patients with other genetic diagnoses without a well established linked to childhood cancer predisposition. **d)** Upset plot summarizing positive ChiCaP criteria in patients that fulfilled at least one criterion (n = 194). The bar charts on top display the number of patients who fulfilled a single criterion (single filled-in dot below the X-axis) or a combination of criteria (filled-in dots connected by lines below the X axis). “Other diagnosis” refers to patients with other genetic diagnoses without a well established linked to childhood cancer predisposition.

For 95% (293/309) of included patients, clinical data were collected through the ChiCaP criteria form (Fig. 3a, Supplementary Table S2). At least one ChiCaP criterion was fulfilled in 66% (194/293) of patients, of which 17% (33/194) had a ChiCaP syndrome (Fig. 3b, Supplementary Table S2). Only two patients were diagnosed with a ChiCaP syndrome by gWGS despite not fulfilling any ChiCaP criteria. These two patients were a girl (BC22) with an IDH-mutant, CNS WHO grade 4 astrocytoma who carried a *de novo* heterozygous germline *MSH2* variant and a boy (BC81) with anaplastic large cell lymphoma and compound heterozygous variants in *FANCM*. The most common reason for fulfilling ChiCaP criteria was the specific malignancy type (45%, 131/293), and half of those patients did not fulfill any additional criterion (Fig. 3d). A positive family history for cancer was reported for 22% (64/290) (Fig. 3d, Supplementary Fig. S3b and d), the most common reason being the occurrence of pediatric cancer in a close family member (10%, 30/290). Cancer in a parent before age 50 years was reported in 4% (12/290) of children.

At least one non-cancer related feature was reported for 30% (88/293) of patients and 9% (26/293) had at least two (Table 1). The most non-cancer related features were cognitive/behavioral disorders (13%, 37/293), skin manifestations (8%, 24/293), and congenital malformations (5%, 15/293) (Supplementary Fig. S3a and c). Intellectual disability or developmental delay was reported in 4% (11/293) (Table 1). When comparing clinical data for patients with or without a ChiCaP syndrome, we confirmed associations with family history of cancer (OR 2.5 95% CI 1.2–5.4) and multiple primary tumors (OR 9.2 95% CI 2.4–35.9). Also the presence of non-cancer related features (“≥1 additional feature” OR 3.9 95% CI 1.9–8.4 and “≥2 additional features” OR 6.4 95% CI 2.5–16.0) and intellectual disability or developmental delay (OR 7.2 95% CI 2.0–25.7) were associated with increased risk for ChiCaP (Table 1). Moreover, the diagnostic yield increased with the number of fulfilled ChiCaP criteria, being 2% (2/100), 10% (11/114), 24% (15/62), and 41% (7/17) for patients fulfilling none, one, two or three ChiCaP criteria, respectively (Fig. 3c).

Seven patients (2%, 7/309) had a previously diagnosed genetic disease without known cancer association (Fig. 3c, Supplementary Table S2). Four out of these seven children had at least two non-cancer related features of which two included intellectual disability (Fig. 3d, Supplementary Fig. S3a–d). Among 29 children with at least two non-cancer related features and/or intellectual disability, eleven (38%, 11/29) had an underlying genetic disorder, either a ChiCaP syndrome or another genetic disease.

### Clinical utility of germline findings

Twenty-four patients (69%, 24/35) received a ChiCaP diagnosis within the study, while eleven patients were diagnosed before tumor diagnosis. For the latter, the study findings corroborated previous clinical recommendations. For the 24 patients diagnosed within the study, all findings were potentially actionable. A direct clinical impact for patient care of the ChiCaP finding was observed in 22 (7% of the whole cohort, 22/298) in terms of either surveillance recommendations (7% of the whole cohort, 21/298) and/or treatment adjustments (3% of the whole cohort, 8/298) during the study period (Fig. 2a, Supplementary Table S3). For example, patient BC308 with nephroblastoma and a germline *FBXW7* variant (OMIM #620012, “Developmental delay, hypotonia, and impaired language”) was planned for prolonged surveillance after completed treatment and a neuropsychiatric assessment due to the previously reported occurrence of neurodevelopmental disorders in patients with germline *FBXW7* variants.^25^ For patient BC156, diagnosed with Burkitt lymphoma and carrying a hemizygous intragenic deletion of *SH2D1A* (diagnostic for X-linked lymphoproliferative syndrome type 1, OMIM #308240), immunological surveillance was initiated and allogeneic stem cell transplantation was planned as further treatment due to risk for hemophagocytic lymphohistiocytosis and for a new or second malignancy. Our cohort included three patients with CMMRD and one patient with CMMRD-like syndrome due to a germline *POLE* variant. All four patients were recommended surveillance and were eligible for immunotherapy, which is ongoing for three patients at the time of writing (Supplementary Table S3). Targeted therapy (Nivolumab and Trametinib later switched to Ivosidenib) was also started for BC25, a girl with a primary MMR-deficient IDH-mutant astrocytoma due to a heterozygous germline *MSH2* variant. All patients were recommended genetic counselling and carrier testing, starting with the parents, when relevant. Given that 28 ChiCaP diagnoses found in our cohort are autosomal dominant, with up to 50% risk of transmission to future generations, our findings are also informative for patients’ future reproductive choices. One additional finding was made during the study. In patient BC228, who was diagnosed with an embryonal rhabdomyosarcoma on the basis of ovarian teratoma and carried germline pathogenic variant in *DICER1,* we also identified a 4.6 Mb large *de novo* deletion on 13q encompassing the genes *BRCA2* and *NBEA*. The deletion likely explains the patient’s intellectual disability and confers increased risk for breast and ovarian cancer in adulthood.

### Somatic analyses to refine sibling risk

In children diagnosed with cancer but without germline findings, the question whether siblings may too be at risk for cancer is often raised. That is especially true for disorders with a high likelihood for germline predisposition such as retinoblastoma. Indeed, siblings to children with retinoblastoma without detected germline *RB1* mutation have a low, but not negligible, risk of retinoblastoma and are usually offered eye surveillance in general anesthesia.^26^ The risk depends on the failure to identify all germline mutations and the possibility of gonadal mosaicism in parents, both of which can be excluded if two somatic hits in *RB1* are identified in tumor DNA and not in blood, thus leaving siblings with population risk only. In our cohort, six patients had unilateral retinoblastoma without germline *RB1* variants (Supplementary Table S5). An additional four patients diagnosed with retinoblastoma between December 2022 and July 2023, without germline *RB1* findings, were included in this analysis. Tumor data (WGS n = 6; WES FFPE n = 3) was available for 90% (9/10) of these patients. Two somatic hits in *RB1* were detected in 78% (7/9) of patients (Supplementary Table S5). In these seven families, siblings were therefore considered to have population risk and could be excluded from surveillance.

## Discussion

In this study, conducted within a Swedish national infrastructure for precision medicine, we combine germline and tumor WGS with systematic phenotyping to study childhood cancer predisposition in a nationwide and prospective cohort of 309 children with CNS and extracranial solid tumors.

Overall, the prevalence of ChiCaP syndrome is this cohort was 11% (95% CI 8%–15%), with a 8% diagnostic yield (24/298, 95% CI 5%–12%) for ChiCaP syndromes not known at diagnosis which were discovered through analysis of gWGS with an in-silico 189-genes panel. These numbers compare well to the 10% prevalence recently reported by nationwide Danish gWGS-based studies both across childhood cancers and in CNS tumors only and to a large study on pediatric CNS tumors.^9^^,^^20^^,^^27^ We applied a stringent definition of a positive germline finding, which needed to fulfill both variant pathogenicity criteria and the syndrome’s known inheritance pattern. This is reflected in a higher concordance of our germline findings with the patients’ clinical phenotypes and the tumors’ somatic profiles compared to the results of previous studies. Fiala et al. reported a diagnostic yield of 13% for variants in moderate and high penetrance dominant genes. Yet, a third of the findings were unexpected based on the patient’s diagnosis and previous history, and majority of those did not show a concordant tumor genotype.^10^ In another study Newman et al. demonstrated a diagnostic yield of 18%, where only about half of the identified P/LP germline variants were labeled as disease-related when reviewed in light of the tumors’ somatic profiles.^4^ While it is expected that the spectrum of tumor types associated with known ChiCaP syndromes might broaden as new evidence is generated, disclosing uncertain germline findings in clinical settings has so far been considered to have limited clinical value.

Nearly all ChiCaP findings from this study provided valuable information to the treating physicians in terms of surveillance or treatment choices. When considering only patients without a known ChiCaP diagnosis, this translates to about 7% (22/298, 95% CI 5%–11%) of children with CNS or extracranial solid tumor having germline findings with potential implication for their surveillance or treatment. Altogheter, 42% (10/24) of ChiCaP diagnoses that were unknown prior to tumor diagnosis followed inheritance patterns consistent with high-risk (25%–50%) for potential siblings to be carriers, thus highlighting the utility of such a finding at the family level. About 2% (6/309) of all patients and as many as 25% (4/16) of patients with high-grade astrocytomas carried germline mutations in MMR genes, which for some patients meant that immunotherapy could be initiated. Due to effective surveillance results in Lynch syndrome and promising data on patients with CMMRD,^28^ having received a ChiCaP diagnosis can lead to increased survival due to presymptomatic detection of new primary tumors. However, due to limited knowledge on the impact of early tumor detection on survival and penetrance for most ChiCaP syndromes, more studies are needed to fully elucidate the clinical short- and long-term impact of germline findings in children with cancer.

The use of systematic phenotyping together with WGS allowed us to compare phenotype-first and genotype-first approaches within the same cohort. Nearly two-thirds of children in our study fulfilled at last one ChiCaP criterion. This proportion is doubled compared to when Jongmans criteria or the McGill Interactive Pediatric OncoGenetic Guidelines (MIPOGG) are used in other studies^9^^,^^12^ and is explained by a broader definition of positive family history and a longer list of specific malignancies used in our study in order to facilitate variant interpretation rather than selection of who to test. However, two patients (BC22 and BC81) diagnosed with ChiCaP syndromes through the study had no positive ChiCaP criteria and would have escaped detection if only a phenotype-first approach had been used. Observations from our cohort, such as the high prevalence of ChiCaP syndromes among patients with high-grade astrocytomas, which corroborates recent results,^20^ and the low prevalence of ChiCaP syndromes among other malignancy types, such as malignant bone tumors, can be used to refine phenotype-first strategies and improve detection of ChiCaP syndromes in low- and middle-income countries where only phenotype-first approaches may be feasible.

Previous studies have shown the value of tumor sequencing in providing diagnostic, prognostic, and therapeutic information.^3^^,^^4^^,^^6^^,^^16^ Overall, our results support the utility of parallel investigations of germline and tumor DNA to establish causality between the detected germline variants and the child’s tumor through analysis of somatic second hits, TMB, and mutational signatures. This is especially feasible in centers that already employ paired tumor-normal analyses for somatic investigations. Similarly, in those centers, the cost of ChiCaP diagnostics is lower since it does not require new sequencing, but only bioinformatics reanalysis. As illustrated for patients with retinoblastoma and no *RB1* germline variants, tumor sequencing data can also be used to refine surveillance recommendations for siblings and future children. In line with the results from previous studies,^29^, ^30^, ^31^ tumor sequencing detected biallelic somatic mutations in *RB1* in 78% (7/9) of patients, whose siblings can be exempted from surveillance, thus sparing them 13 examinations under anesthesia during their first four years of life. A similar approach can be used for other tumors that are often hereditary, such pleuropulmonary blastoma caused by *DICER1* mutations. Due to remaining risk for somatic mosaicism, tumor sequencing followed by targeted analysis in blood could be a better strategy to assess risk for second generations. Depending on local organization of the diagnostic services, it may be that germline and somatic analyses are performed at different departments, as was the case in this study. In these settings, discussions at multidisciplinary molecular tumor boards help to integrate results from germline and tumor analyses.

WGS is emerging as the preferred sequencing method for precision diagnostics due to its ability to reliably detect multiple types of variants, including SVs,^15^^,^^16^^,^^32^ which accounted for 16% of the findings in our cohort. The same advantage with gWGS applies to tumor WGS, also due to the frequency of SVs and gene fusions in childhood cancer.^4^^,^^16^ On the other hand, gWGS provides lower sequencing depth than exome or targeted panels and is therefore less suitable to detect low-grade mosaic variants. In patients with strong clinical suspicion of a specific ChiCaP syndrome, other methods such as RNA-sequencing for detection of splicing aberrations and deep DNA-sequencing for detection of mosaicism may be better and cheaper choices. All short-read sequencing methods would fail to detect ChiCaP syndromes caused by methylation defects such as some forms of Beckwith-Wiedemann syndrome for which phenotype-guided diagnostics is still gold standard.

Another limitation of the study is the underepresentation of CNS tumor and lymphomas. Morever, despite the national scale, the cohort size together with the broad heterogeneity of tumor types included affected our power for subgroup analyses pertaining to specific tumor types or patients with distinct clinical features, whose results require confirmations in larger cohorts. Strengths of this study are the prospective nature and the combined phenotype-genotype-driven approach in a unselected cohort of pediatric patients with solid tumors.

Overall, our results demonstrate that genomics-based diagnostics of ChiCaP in children with solid tumors is feasible in large-scale clinical practice and critically guides personalized care in a sizable proportion of patients. In clinical settings, we advocate for stringent germline variant interpretation, which may be supported by parallel analysis of tumor sequencing data if available. Systematic phenotyping is important for genotype–phenotype correlations and to identify patients with high suspicion of ChiCaP syndrome or other genetic disorders for which extended germline testing is warranted.

## Contributors

Conceptualization; Ann Nordgren, Fulya Taylan, Bianca Tesi, David Gisselsson, Gustaf Ljungman, Emma Tham. Data curation: Bianca Tesi, Kristina Lagerstedt Robinson, Frida Abel, Teresita Díaz de Ståhl, Sara Orrsjö, Anna Poluha, Maria Hellberg, Sandra Wessman, Sofie Samuelsson, Tony Frisk, Hartmut Vogt, Karin Henning, Magnus Sabel, Per Nyman, Geraldine Giraud, Joakim Wille, Cornelis Jan Pronk, Ulrika Norén-Nyström, Magnus Borssén, Maria Fili, Linda Arvidsson, Anna Rosén, Ekaterina Kuchinskaya, Anna-Lotta Hallbeck, Gunilla Kanter Smoler, Alia Shamik, Fredrik Mertens, Emma Tham, Pernilla Grillner, Gustaf Ljungman, David Gisselsson, Fulya Taylan, Ann Nordgren. Formal analysis: Bianca Tesi, Kristina Lagerstedt Robinson, Frida Abel, Teresita Díaz de Ståhl, Sara Orrsjö, Anna Poluha, Maria Hellberg, Sandra Wessman, Sofie Samuelsson, Torben Ek, Carolina Maya-Gonzalez, Linda Arvidsson, Ekaterina Kuchinskaya, Gunilla Kanter Smoler, Giorgio Tettamanti, Päivi Lähteenmäki, Susanne Fransson, Tommy Martinsson, Alia Shamik, Fredrik Mertens, Emma Tham, David Gisselsson, Fulya Taylan, Ann Nordgren. Funding acquisition: Ann Nordgren, David Gisselsson, Gustaf Ljungman, Johanna Sandgren, Richard Rosenquist. Investigation: Bianca Tesi, Kristina Lagerstedt Robinson, Frida Abel, Teresita Díaz de Ståhl, Sara Orrsjö, Anna Poluha, Maria Hellberg, Sandra Wessman, Sofie Samuelsson, Tony Frisk, Hartmut Vogt, Karin Henning, Magnus Sabel, Torben Ek, Niklas Pal, Per Nyman, Geraldine Giraud, Joakim Wille, Cornelis Jan Pronk, Ulrika Norén-Nyström, Magnus Borssén, Maria Fili, Gustav Stålhammar, Nikolas Herold, Giorgio Tettamanti, Carolina Maya-Gonzalez, Linda Arvidsson, Anna Rosén, Katja Ekholm, Ekaterina Kuchinskaya, Anna-Lotta Hallbeck, Margareta Nordling, Pia Palmebäck, Per Kogner, Gunilla Kanter Smoler, Päivi Lähteenmäki, Susanne Fransson, Tommy Martinsson, Alia Shamik, Fredrik Mertens, Emma Tham, Pernilla Grillner, Gustaf Ljungman, David Gisselsson, Fulya Taylan, Ann Nordgren. Project administration: Bianca Tesi, Kristina Lagerstedt Robinson, Frida Abel, Teresita Díaz de Ståhl, Sara Orrsjö, Anna Poluha, Maria Hellberg, Sandra Wessman, Sofie Samuelsson, Tony Frisk, Hartmut Vogt, Karin Henning, Magnus Sabel, Per Nyman, Geraldine Giraud, Joakim Wille, Cornelis Jan Pronk, Ulrika Norén-Nyström, Magnus Borssén, Maria Fili, Gustav Stålhammar, Linda Arvidsson, Margareta Nordling, Pia Palmebäck, Gunilla Kanter Smoler, Alia Shamik, Fredrik Mertens, Valtteri Wirta, Emma Tham, Pernilla Grillner, Johanna Sandgren, Gustaf Ljungman, David Gisselsson, Fulya Taylan, Ann Nordgren. Software: Valtteri Wirta, Teresita Díaz de Ståhl. Supervision: Ann Nordgren, Fulya Taylan, David Gisselsson, Gustaf Ljungman. Visualization: Bianca Tesi, Fulya Taylan. Writing—original draft: Bianca Tesi, Fulya Taylan, Ann Nordgren. Writing—review & editing: all authors.

## Data sharing statement

Raw and analyzed genomic data from the study have been deposited at the Swedish Childhood Tumor Biobank and are available for secondary use within research projects on pediatric cancer through a controlled data access procedure. This procedure is also described in the study consent signed by all study subjects and/or parents or legal guardians.

## Declaration of interests

BT, FA, ET, and AN received support from the Swedish Childhood Cancer Fund (BT: TJ2018-0042; FA: KP2021-0010; ET: TJ2021-0125; AN: KP2019-0024, PR2019-0027, TJ2019-0013) and the Swedish Cancer Fund (FA: 21 1540 Fk 01 H; ET: 22 2451Fk; AN: 22 2057Pj). BT, ET and AN received support from Region Stockholm (BT: FoUI-985957; ET: FoUI-973659; AN: 5010124 ALF, 520136 ALF). AN received support from The Swedish Research Council (2021-02860). MB received honoraria for lectures by the Swedish Childhood Cancer Fund. GS served as advisor for trial design for Cyxone AB, Sweden. NH served as Chair of NOPHO Scientific Committee and Young NOPHO without retribution. RR received honoraria from AbbVie, AstraZeneca, Janssen, Illumina, and Roche. DG received grants from Swedish Ministry of Health and Social Affairs for GMS Childhood Cancer and is Vice dean for internationalization and recruitment, Faculty of Medicine, Lund University. AN received also funding from the Cancer Society of Stockholm, Stiftelsen Frimurare Barnhuset i Stockholm, Hållsten research foundation, Berth von Kantzow foundation and is board member of Sävstaholm foundation, Ågrenska foundation, Sällsyntafonden. All other authors have no conflict of interest to declare.
