# Supplementary material for: Diagnostic yield and clinical impact of germline sequencing in children with CNS and extracranial solid tumors—a nationwide, prospective Swedish study
Source: Lancet Reg Health Eur. 2024 Mar 19;39:100881. doi: 10.1016/j.lanepe.2024.100881 (PMC11129334; doi:10.1016/j.lanepe.2024.100881)
Supplement: Supplementary Material [file mmc2.pdf]

Supplementary data – Table of contents

Supplementary methods ... **1-11**

Germline whole genome sequencing ... 1

Tumor whole genome sequencing ... 2

Chicap gene list ... 3

Chicap criteria form ... 3

Analysis of data Chicap criteria form ... 8

Comparison with Swedish Childhood Cancer Registry data ... 9

References ... 10

Supplementary figures ... **12-15**

Supplementary figure legends ... 12

Supplementary figure 1 ... 13

Supplementary figure 2 ... 14

Supplementary figure 3 ... 15

Supplementary tables ... **16-20**

Supplementary table 1 ... 16-19

Supplementary table 5 ... 20

Supplementary methods

Germline whole genome sequencing

Germline whole genome sequencing (gWGS) was performed starting with 1 μg DNA extracted from the patient's normal blood lymphocytes (germline). Sequencing was performed as paired-end 150-bp readout mode on S4 flow cells using the NovaSeq 6000 sequencer (Illumina). gWGS data was analyzed through locally established germline pipelines at the three participating clinical genetics departments. All centers filtered the data using a Childhood Cancer Predisposition (ChiCaP) gene list emcopassing 189 genes (Supplementary table 1). The gene list is described more in detailed below. Specific differences in the bioinformatic pipelines including variant prioritization are outlined below.

However, all centers employed the same strategy for which variants to be reported. Variants were classified according to guidelines from the American College of Medical Genetics (ACMG).[1] Pathogenic and likely pathogenic variants scored as ACMG class 4 (likely pathogenic, LP) and 5 (pathogenic, P) were reported if: 1) they fitted the known inheritance pattern for the specific ChiCaP syndrome (e.g. biallelic P/LP variants in *ATM*) or constituted heterozygous P/LP variants in genes causative of high-risk dominant adult-onset cancer predisposition syndrome (e.g. *BRCA1/2*, Lynch syndrome, *PTEN,* etc.). Heterozygous variants in moderate-risk genes for adult-onset cancers (e.g. heterozygous P/LP variant in *ATM*) were not reported. Variants of uncertain significance defined as ACMG class 3 were reported only when both the variant characteristics indicated pathogenicity (i.e., not present in normal variation databases, high local ranking, compound variant present for autosomal recessive genes) and the patients´ phenotype had clinical presentation in line with the phenotype described for the affected gene. In these case, segregation analysis with parental samples was performed – e.g., to prove *de novo* origin of the variant – prior to variant classification as P/LP. All cases were analyzed as singletons.

Analysis of gWGS performed at the Department of Clinical Genetics of the Karolinska University Hospital (for samples from Stockholm, Linköping, Umeå and Uppsala) was carried out using the Mutation Identification Pipeline framework (MIP) pipeline (v.9.1.3 to 11.2.2), a standard of care procedure for detecting rare germline variants (single nucleotide variants – SNVs – and indels and structural variants, SVs) in use at the Genomic Medical Center Karolinska, as described previously.[2] Variants are ranked through weighting of different annotations to help variant prioritization, as previously described.[3] During the study time SNVs rank model v.1.31 to 1.34 and SV rank model v.1.8 to v.1.9 were used. Annotated and ranked variants were further interpreted using the visualization tool Scout (version 4.36 or higher; https://github.com/Clinical-Genomics/scout.) The hg19 (GRCh37) genome assembly was used for reference.

Analysis of gWGS performed at the Department of Clinical Genetics and Genomics, Sahlgrenska University Hospital, Gothenburg (for samples from Gothenburg) was carried out using the Sentieon bioinformatical tool DNAscope (Sentieon version: v201911 and v202112) for calling of SNVs and small indels, and calling of copy number variants (CNV) using Canvas (version 1.40.0.1613, Illumina), as described previously.[2] Variants (SNV and CNV) were filtered in Alissa Interpret (version 5.3.3, www.agilent.com) keeping known likely pathogenic or pathogenic variants (according to HGMD professional https://portal.biobase-international.com and/or ClinVar https://www.ncbi.nlm.nih.gov/clinvar/ mutation databases), or non-synonymous and/or potential splice-site variant not present in the normal population databases (gnomAD; 1000 genomes; ExAC) above 5%. Annotated and filtered variants were further interpreted using the

Alamut Visual (https://www.sophiagenetics.com/platform/alamut-visual-plus). The hg38 (GRCh38) genome assembly was used for reference.

Analysis of gWGS performed at Department of Clinical Genetics, Pathology and Molecular Diagnostics, Region Skåne, Lund, was carried out using the pipeline in use for diagnostics of rare diseases, as previously described.[2] Briefly, SNVs and indels were called according to Sentieon DNAscope (202112) best practice workflow. The scope of SNV/ indel calling was defined by a BED file; variants in coding sequence and +/- 20 bp up- and downstream were called, as were variants in non-coding sequences at the time described as pathogenic or likely pathogenic in ClinVar. Detected SNVs and indels were annotated with information from VEP, vcfanno, CADD, ClinVar and Genmod (https://github.com/moonso/genmod). SV calling was performed by three different variant callers; GATK, TIDDIT, and Manta. Variants identified by more than one caller, with a minimum overlap of 70% , were merged by a local software. Detected SVs were annotated with information from VEP, AnnotSV, Pre-score (local Pearlscript), Genmod and Compound finder (local Pearlscript). Variants (SNVs, indels, SVs) were then scored and ranked based on the attributed information. The resulting vcf-files were uploaded to Scout for clinical interpretation and documentation. Prioritized variant-lists were assessed manually in Scout, with support from Alamut (https://www.sophiagenetics.com/platform/alamut-visual-plus), IGV and locally developed visualization tools (e.g. Gens). Variants within the current gene panel exceeding a set rank score cut-off were interpreted. The hg38 (GRCh38) genome assembly was used for reference.

Tumor whole genome sequencing

Tumor WGS was performed starting with 1 µg DNA extracted from fresh or frozen tumor tissue (somatic) when available. Sequencing was performed as paired-end 150-bp readout mode on S4 flow cells using the NovaSeq 6000 sequencer (Illumina) as described in the main text. Specific differences in the bioinformatic pipelines among centers are outlined below.

At Clinical Genomics, Karolinska Institutet, Stockholm analysis of tumor WGS (tissue samples from Linköping, Stockholm, Umeå and Uppsala) was carried out using a custom-developed bioinformatic pipeline called Balsamic (version 7.2.2 or higher; https://balsamic.readthedocs.io/en/latest/index.html). The pipeline utilizes Sentieon bwa-mem for alignment, TNScope (Sentieon) for calling of SNVs and indels, ascatngs for calling of copy number alterations (CNAs) and Manta for SV calling. The reference genome hg19 was used. Variants were annotated using VEP. SNV and indel variants fulfilling the following criteria were passed: total read depth >=10, alternative allele read depth >=3, VAF >=0.05 and <1.

At the Department of Clinical Genetics and Genomics, Sahlgrenska University Hospital, Gothenburg analysis of tumor WGS (tissue samples from Gothenburg) was carried out using a custom-developed bioinformatic pipeline called "WGS Somatic" utilizing TNscope (Sentieon) for calling of somatic SNVs and indels, Canvas for calling of CNAs and Manta for SV calling, as described previously (PMID: 37384868). The reference genome hg38 (GRCh38) was used for assembly. Somatic SNV/indel filtering was performed using QIAGEN Clinical Insight Interpret (version 8.1.20210827; https://digitalinsights.qiagen.com) keeping only non-synonymous and potential splice-site variants with total read depth >=10, VAF >=0.10. Filtering of somatic SVs was performed according to the following criteria: supportive reads >=3 sample supported by both paired and split spanning reads in the tumor, and allowing maximum 1 supportive reads in the normal sample.

At the Department of Clinical Genetics, Pathology and Molecular Diagnostics, Region Skåne, Lund (tissue samples from Lund) analysis of tumor WGS was carried out using an in-house nextflow based bioinformatic pipeline implemented for identification of somatic variations from paired tumor/normal cancer samples, as previously described.[2] Briefly, short sequencing reads were assembled to hg38 using Sentieon distributed bwa-mem mode (202112.05). Subsequently, somatic SNVs and indels were identified with somatic variant caller tools – freebayes and VarDict. Additionally, somatic CNAs were identified using the GATK copy number variant module. Lastly, SV including gene fusions were identified with Manta Structural Variant Caller tool (MantaSV, v1.6.0, Illumina) and annotated with the SnpEFF toolkit. All somatic variants (SNVs, indels, CNAs, and SVs including fusion genes) were interpretated, annotated, and compared to information from public databases, using an inhouse software called Coyote. All the selected variants were manually inspected in the IGV or the locally developed visualization tools called Gens before further classification according to their clinical impact.

ChiCaP gene list

Germline whole genome sequencing (gWGS data) were filtered with the help of a gene list emcompassing 189 genes (Supplementary table 1). The gene list was produced through review of available gene lists[4–6]. Fifty-five genes on the list were considered actionable because pathogenic variants were either linked to established recommendations for surveillance or as they likely to result in treatment adjustments in children with cancer regardless of diagnosis. Actionability was assessed based on the American College of Medical Genetics' list of secondary findings v.2[7]; the ClinGen Pediatric actionable gene list (https://actionability.clinicalgenome.org/ac/ last accessed 2021-03-02); available surveillance program in either national guidelines or in well-established international literature prior to study start[8]. The remaining 134 genes were also associated with known ChiCaP syndromes prior to study start but their potential impact on cancer care was less well established. Gene lists were discussed and approved by multidisciplinary groups before study start. For disorders with high genetic heterogeneity, e.g. Fanconi anemia, all genes associated with the disorders were included even though the evidence for causality was in some cases only based on reports from 1-2 families.

ChiCaP criteria form

At inclusion, information on malignancy type, clinical features suggestive of ChiCaP, and family history of cancer was collected through the ChiCaP criteria form. Translation of the ChiCaP criteria form from Swedish to English was done using DeepL (https://www.deepl.com/en/translator), and is provided below. The form was built upon existing clinical screening tools such as Jongmans criteria, MIPOGG, etc.[5,9–11]. Focus was given to clinical features that could be assessed at diagnosis. The form collects information pertinent to four domains: 1) family history of cancer and/or parental consanguinity; 2) cancer history in the child in terms of multiple primary malignancies and/or bilateral tumors; 3) "tip-to-toe" review of non-cancer related features; 4) specific tumor diagnosis with higher prior likelihood of underlying predisposition. A box for comments in free text was available at the end of the form for the physicians to provide further information. Domains 1, 2 and 4 were designed after a comparison of existing screening tools to create a combined list[5,9–11]. Therefore, by design, the ChiCaP criteria form was expected to turn out positive in a larger proportion of children compared to other tools. The ChiCaP criteria form was filled out by physicians specialized in pediatric, pediatric oncology

or medical oncology shortly after the collection of informed consent from parents or legal guardians.

1. **Family history:** *Ask about cancer in at least three generations i.e. parents, siblings, parents' siblings, cousins, grandparents, grandparents' siblings, grandparents, grandparents' siblings, possibly other relatives. Mark with a cross.*

   ☐ The child's parents are related.

   ☐ At least one relative with cancer before age 18.

   ☐ A parent or sibling/half-sibling affected by cancer before the age of 50.

   ☐ At least 2 relatives on the same side of the family with cancer before the age of 50.

   ☐ At least one relative with multiple cancers.

   ☐ At least one relative with the same type of cancer/tumor as the child.

   Indicate which relative, if on mother's/father's side and type of cancer

   ☐ None of the above

2. **Child's medical history of malignancy/tumor:** *Mark with a cross.*

   ☐ > 1 primary malignancy/tumor (synchronous - within 6 months or metachronous - after 6 months) regardless of diagnosis.

   ☐ Bilateral or multifocal primary malignancy/tumor regardless of diagnosis

   ☐ None of the above

3. **Congenital malformations or other specific symptoms.** *Mark with a cross.*

**Category**		
**Cognition / Behavior**	☐ Age-appropriate, without remarks<br>☐ Intellectual disability<br>☐ Development delay<br>☐ Autism spectrum disorder	☐ Other:

**Abnormal growth* (*If abnormal, attach growth charts)**	☐ Growth and proportions, etc.<br>☐ Small for gestational age (SGA) height or weight < -2 SDS for gestational length<br>☐ Short-grown (< -2.5 SD)<br>☐ Tall (> 2.5 SD)<br>☐ Macrocephaly (> 2 SD)<br>☐ Microcephaly (< -2 SD)<br>☐ Obesity (Iso-BMI > 30)<br>☐ Abnormal body proportions	☐ Wide neck<br>☐ hemihypertrophy<br>☐ Macroglossia<br>☐ Other asymmetry<br>☐ Other anomaly:
**Facial dysmorphology**	☐ None<br>☐ Clear suspicion<br>☐ Some suspicion	☐ In case of suspicion, try to describe what is involved:
**Congenital malformations/ Other abnormalities**	☐ No malformations/other abnormalities<br>☐ Congenital severe visual impairment<br>☐ Congenital severe hearing loss<br>☐ Cleft lip and palate<br>☐ Internal malformations (heart, vessels, CNS, kidneys, GI, airways)<br>☐ Skeletal dysplasia/skeletal malformation<br>☐ Abnormal shape of the chest<br>☐ Radial anomalies including thumbs<br>☐ Other limb abnormalities<br>☐ Craniosynostosis<br>☐ Abnormal urogenitalia	☐ Strabismus<br>☐ Glaucoma/Cataract<br>☐ Aniridia<br>☐ White pupil reflex<br>☐ Other eye malformations<br>☐ Cysts in internal organs<br>☐ Other anomaly:
**Hair and skin**	☐ Unremarkable<br>☐ >2 café-au-lait spots<br>☐ Hypo/hyper-pigmentation<br>☐ Freckles on an unusual area (e.g. lips, inside the mouth, on the glans, on fingers and toes, axillae or groin).<br>☐ Teleangiectasia<br>☐ Other vascular skin changes<br>☐ Multiple benign skin tumours<br>☐ Hairy skin plaques<br>☐ Hypersensitivity to sunlight	☐ Brittle / grey hair<br>☐ Abnormal hairiness<br>☐ Brittle nails<br>☐ Leukoplakia<br>☐ Pit / fold in ear helix<br>☐ Supernumerary nipple<br>☐ Other anomaly:

**Neurological/ Muscular/ Connective tissue abnormalities (not due to cancer)**	☐ No neurological / muscular / connective tissue abnormalities<br>☐ Ataxia<br>☐ Epilepsy<br>☐ Spasticity<br>☐ Muscular hypotonia	☐ Dystonia<br>☐ hypermobility<br>☐ Vascular emergencies<br>☐ Hernia<br>☐ Other anomaly:
**Hematological abnormalities (not due to the cancer)**	☐ No hematological abnormalities<br>☐ Pancytopenia<br>☐ Anemia<br>☐ Thrombocytopenia<br>☐ Neutropenia	☐ Leukopenia<br>☐ Macrocytic erythrocytes<br>☐ Other anomaly:
**Immune system**	☐ No immunological defects<br>☐ Investigated for immune deficiency<br>☐ Hypogammaglobulinemia<br>☐ Autoimmunity	☐ Inflammation<br>☐ Other anomaly:
**Endocrine abnormalities**	☐ No endocrine abnormalities<br>☐ Primary hyperparathyroidism<br>☐ Early/late puberty<br>☐ Hypogonadism<br>☐ Prolactinoma<br>☐ Gigantism / acromegaly	☐ Cushing's syndrome<br>☐ Goiter<br>☐ Other anomaly:

☐ none of the above congenital malformations or other specific symptoms

1. **Please indicate below the child's confirmed/suspected cancer/tumor *diagnosis:***

☐ Pathology report not ready - suspected diagnosis: ______________________________

☐ The child has one of the following diagnoses. *Check the box if the diagnosis has been established.*

Any of the following malignancies/tumours of the nervous system:	☐ Atypical teratoid rhabdoid tumor<br>☐ Dysplastic cerebellar gangliocytoma<br>☐ Endolymphatic sac tumors<br>☐ Ependymoma<br>☐ Hemangioblastoma<br>☐ Pituitary gland: adenoma/tumor/blastoma<br>☐ Malignant/benign peripheral nerve sheath tumor<br>☐ Medulloblastoma<br>☐ Medulloepithelioma

	☐ Meningioma<br>☐ Optic glioma<br>☐ Pineoblastoma<br>☐ Choroidal plexus carcinoma<br>☐ Retinoblastoma<br>☐ Schwannomas (vestibular schwannomas or schwannomatosis)<br>☐ Subependymal giant cell astrocytoma	
Any of the following solid tumors	☐ Angiomyolipoma (kidneys and/or liver)<br>☐ Adrenalcortical carcinoma<br>☐ Cystic nephroma<br>☐ Fetal rhabdomyoma<br>☐ Gastrointestinal stromal tumor<br>☐ Gonadoblastoma<br>☐ Hepatoblastoma<br>☐ Infantile myofibromatosis<br>☐ Chondromesenchymal harmatoma<br>☐ Gonadal stromal cell tumor with annular tubules<br>☐ Myxoma<br>☐ Neuroblastoma<br>☐ Neuroendocrine tumors<br>☐ Paraganglioma/Pheochromocytoma<br>☐ Parathyroid cancer/adenoma<br>☐ Pleuropulmonary blastoma<br>☐ Rhabdoid tumor (RT): Atypical teratoid RT or renal / extrarenal malignant RT<br>☐ Rhabdomyosarcoma<br>☐ Sertoli-Leydig cell tumor<br>☐ Small cell ovarian cancer of hypercalcemic type<br>☐ Wilms' tumor	
Any cancer usually seen in adults:	☐ Basalioma<br>☐ Colorectal cancer<br>☐ Liver cancer<br>☐ Malignant melanoma<br>☐ Kidney cancer	☐ Ovarian cancer<br>☐ Squamous cell carcinoma<br>☐ Thyroid cancer<br>☐ Other tumor:
Any other benign tumor that may indicate cancer predisposition syndrome	☐ Angiofibroma<br>☐ Fibrofolliculoma<br>☐ Keratocystic odontogenic tumor<br>☐ Myofibromatosis	☐ Neurofibroma<br>☐ Trichodiscoma<br>☐ Other tumor:

☐ None of the above confirmed / suspected cancer / tumor diagnosis

Assessment of family history of cancer was facilitated through a questionnaire that could be filled out by the child's parents. This questionnaire was optional. The following questions were asked:

1) Is there any relationship between the two of you? (For example, are you cousins, second cousins or do you know that you are distantly related to each other?) If yes - please describe how you are related;
2) Have any of you had cancer? If yes - please indicate which of you has had cancer and what type of cancer and at what age;
3) Do you have other children (from the same or different relationship) who have had cancer in childhood or adulthood? If yes - please specify who and if possible what type of cancer and at what age;
4) Is there anyone else in your family who got cancer before the age of 18? If yes - please specify who and if possible what type of cancer and at what age;
5) Is there anyone among your parents and siblings who got cancer before the age of 50? If yes - please describe on whose side of the family, how that person is related to you, and, if possible, what type of cancer and at what age;
6) Are there any other relatives with multiple cancers? If yes, please specify who and, if possible, what type of cancer and at what age.

Analysis of data from ChiCaP criteria form

Data collected through the ChiCaP criteria form were manually reviewed and assessed for fulfillment of ChiCaP criteria. The family history criterion (domain 1) was considered fulfilled if any of the following conditions were met:

- Parent_cancer_before_50yo: parent with any type of cancer before age 50 years;
- 2_rel_cancer_before_50yo: 2 relatives with any type of cancer on same side before age 50 years;
- rel_cancer_before_18yo: relative with any type cancer before age 18 years;
- rel_same_organ: relative with cancer affecting the same organ, regardless of age at diagnosis;
- rel_same_cancer: relative with same type of cancer, regardless of age at diagnosis;
- rel_2_cancer_before_60yo: relative with 2 primary malignancies before age 60 (excluding nonmelanoma skin cancer);
- halfsib_cancer_before_50yo: half sibling with any type of cancer before age 50;
- Consanguinity: consanguineous parents (any level).

The criterion for child's medical history of malignancy was considered fulfilled only in children with multiple primary tumors, i.e. included at diagnosis of second/third primary tumor.

The criterion for other symptoms was considered fulfilled if any features were reported that could not be explained by the underlying CNS or extracranial solid tumor. For further analysis, clinical features were grouped as follows (examples of reported clinical features are provided for each category):

- Cutaneous: any reported skin/hair feature – e.g. stork bite, hemangioma, telangiectasia, hypo/hyperpigmentation, cafe-au-lait spots;
- Cognition_behaviour: any reported anomaly of cognition/behaviour – e.g. ADD, ADHD, autism, autism spectrum disorder, intellectual disability, developmental delay, ongoing neuropsychiatric assessment;

- Growth: any reported growth abnormality – e.g. length > 2SD, obesity, short stature, microcephaly;
- Inflammation_immune: any reported immunological defect – e.g. immunodeficiency, Periodic Fever Aphthous Stomatitis Pharyngitis Adenitis, systemic juvenile arthritis;
- Malformation: any reported congenital malformation – e.g. kidney cysts, kidney malformation, umbilical hernia, single testicle, heart malformation, polydactyly;
- Neuro_muscular: any reported neuromuscular symptoms – e.g. hypotonia, epilepsy, tics, motor developmental delay;
- Endocrine: any reported hormonal defect – e.g. diabetes mellitus type 1, abnormal gonadotropine levels;
- Dysmorphism: facial dysmorphism;
- Other: any more rarely reported abnormality, occurring ≤2 in the whole cohort – e.g. hearing loss, blindness, blood abnormality;

The child was classified as having ≥2 features in case of multiple congenital malformations, multiple type of skin changes (eg. pigmentation and vascular), or symptoms/signs affecting >1 system, e.g. cutaneous and growth. The child was classified as having intellectual disability (ID) or developmental delay (DD) only when the respective boxes on the form were ticked. The criterion for malignancy type was considered fulfilled in all children with a final diagnosis listed under that criteria.

Comparison with Swedish Childhood Cancer Registry data

To assess population coverage of the GMS-ChiCaP study we conducted a comparative analysis using data from the Swedish Childhood Cancer Registry (SCCR). The SCCR is a National Quality Registry used for the evaluation of treatment outcomes for children diagnosed with cancer. For children diagnosed with cancer below 15 years of age in Sweden a 100% completeness is expected and was calculated at 93% through a validation project initiated since the fall of 2020[12]. This validation project showed that highest percentage of dropout in SCCR data applied to brain tumors, retinoblastoma, and carcinoma. For the older patient group, the coverage rate was 44% where carcinoma diagnoses account for 47% of the dropout. This can be explained by the fact that not all patients over 16 years at diagnosis are treated at pediatric oncology centers and therefore do not fall within the target population[12]. For the comparison with data from the GMS-ChiCaP study, data on cancer diagnoses registered between 1st May 2021 and 31st December 2022 were extracted. It should be noted that patients were included in the study cohort based on the date when the blood sample for gWGS reached the Department of Clinical Genetics and not on the date of tumor diagnosis, so the cohorts are likely to differ slightly around the beginning and end of the study.

References

1. Richards S, Aziz N, Bale S, Bick D, Das S, Gastier-Foster J, et al. Standards and guidelines for the interpretation of sequence variants: a joint consensus recommendation of the American College of Medical Genetics and Genomics and the Association for Molecular Pathology. Genet Med. 2015 May;17(5):405–24.

2. Wadensten E, Wessman S, Abel F, Diaz De Ståhl T, Tesi B, Orsmark Pietras C, et al. Diagnostic Yield From a Nationwide Implementation of Precision Medicine for all Children With Cancer. JCO Precision Oncology. 2023 Aug;(7):e2300039.

3. Stranneheim H, Lagerstedt-Robinson K, Magnusson M, Kvarnung M, Nilsson D, Lesko N, et al. Integration of whole genome sequencing into a healthcare setting: high diagnostic rates across multiple clinical entities in 3219 rare disease patients. Genome Med. 2021 Mar 17;13(1):40.

4. Byrjalsen A, Diets IJ, Bakhuizen J, Hansen T van O, Schmiegelow K, Gerdes AM, et al. Selection criteria for assembling a pediatric cancer predisposition syndrome gene panel. Familial Cancer. 2021 Oct 1;20(4):279–87.

5. Ripperger T, Bielack SS, Borkhardt A, Brecht IB, Burkhardt B, Calaminus G, et al. Childhood cancer predisposition syndromes—A concise review and recommendations by the Cancer Predisposition Working Group of the Society for Pediatric Oncology and Hematology. American Journal of Medical Genetics Part A. 2017;173(4):1017–37.

6. Wilson CL, Wang Z, Liu Q, Ehrhardt MJ, Mostafavi R, Easton J, et al. Estimated number of adult survivors of childhood cancer in United States with cancer-predisposing germline variants. Pediatr Blood Cancer. 2020;67(2):e28047.

7. Kalia SS, Adelman K, Bale SJ, Chung WK, Eng C, Evans JP, et al. Recommendations for reporting of secondary findings in clinical exome and genome sequencing, 2016 update (ACMG SF v2.0): a policy statement of the American College of Medical Genetics and Genomics. Genetics in Medicine. 2017 Feb 1;19(2):249–55.

8. Brodeur GM, Nichols KE, Plon SE, Schiffman JD, Malkin D. Pediatric Cancer Predisposition and Surveillance: An Overview, and a Tribute to Alfred G. Knudson Jr. Clin Cancer Res. 2017 Jun 1;23(11):e1–5.

9. Jongmans MCJ, Loeffen JLCM, Waanders E, Hoogerbrugge PM, Ligtenberg MJL, Kuiper RP, et al. Recognition of genetic predisposition in pediatric cancer patients: An easy-to-use selection tool. European Journal of Medical Genetics. 2016 Mar 1;59(3):116–25.

10. Goudie C, Coltin H, Witkowski L, Mourad S, Malkin D, Foulkes WD. The McGill Interactive Pediatric OncoGenetic Guidelines: An approach to identifying pediatric oncology patients most likely to benefit from a genetic evaluation. Pediatric Blood & Cancer. 2017;64(8):e26441.

11. Postema FAM, Hopman SMJ, Borgie CAJM de, Hammond P, Hennekam RC, Merks JHM, et al. Validation of a clinical screening instrument for tumour predisposition syndromes in patients with childhood cancer (TuPS): protocol for a prospective, observational, multicentre study. BMJ Open. 2017 Jan 1;7(1):e013237.

12. Swedish Childhood Cancer Registry Annual Report, Barncancerregistret, 2021. [Internet]. Available from: https://sbcr.se/

Supplementary Figure legends

Supplementary Figure 1. a) Age distribution of patients in relation to main ICCC-3 groups. **b)** Treemap of the different categories of CNS tumors (ICCC-3 group III, Xa). HG=high grade; LG=low-grade.“LG astrocytoma” includes glioneuronal tumors. “Other tumor” refers to tumors that do not fit in any of the other groups.

Supplementary Figure 2. a) Number of patients without known ChiCaP diagnosis at inclusion who received a molecular diagnosis of ChiCaP through the study in relation to ICCC-3 groups. CNS tumors emcompass both group III and group Xa. Nb=neuroblastoma; Rb=retinoblastoma; GCT=germ cell tumor. **b)** Number of patients without known ChiCaP diagnosis at inclusion who received a molecular diagnosis of ChiCaP through the study in relation to different categories of CNS tumors. HG=high-grade; LG=low-grade. **c)** Histogram of mutated genes among patients who received a molecular diagnosis of ChiCaP through the study in relation to main ICCC-3 groups.

Supplementary Figure 3. a) Upset plot showing detail of non-cancer related symptoms in patients with at least one non-cancer related features (n=88). Features are explained in supplementary methods. **b)** Upset plot showing family history for cancer in patients who fulfilled the ChiCaP family history criteria (n=64). The different categories for family history are explained in supplementary methods. In a and b the bar charts on top display the number of patients who fulfilled a single feature or family history category (single filled-in dot below the X-axis) or a combination of thoses (filled-in dots connected by lines below the X-axis). **c)** Distribution of non-cancer related features in children diagnosed with CNS amd extracranial solid tumor. **d)** Distribution of positive family history for cancer in children diagnosed with CNS and extracranial solid tumor. All barcharts are colored according to germline status where “Other diagnosis” refers to patients with other genetic diagnoses without a well-established linked to childhood cancer already known at study inclusion.

a

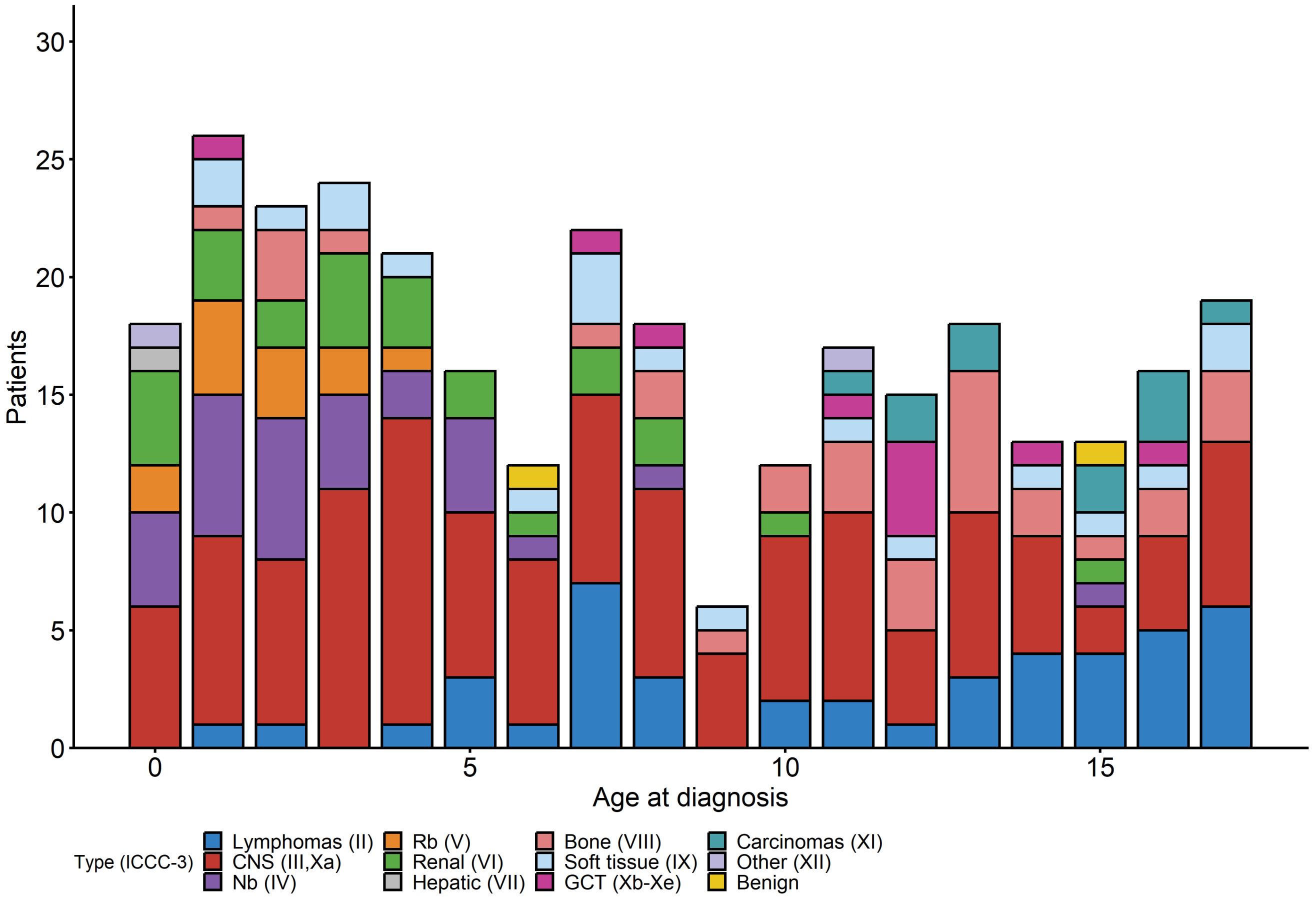

b

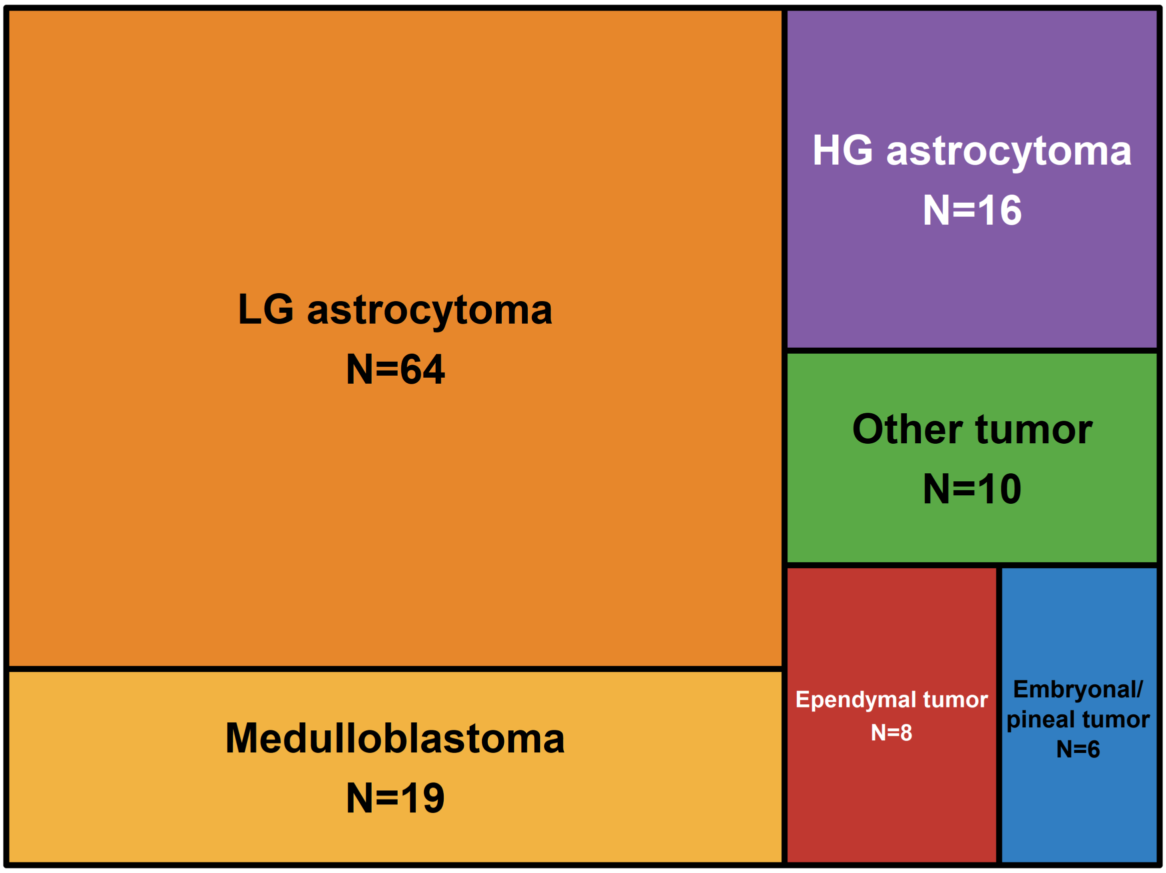

Supplementary Figure 1. Tesi, Lagerstedt Robinson, Abel, et al.

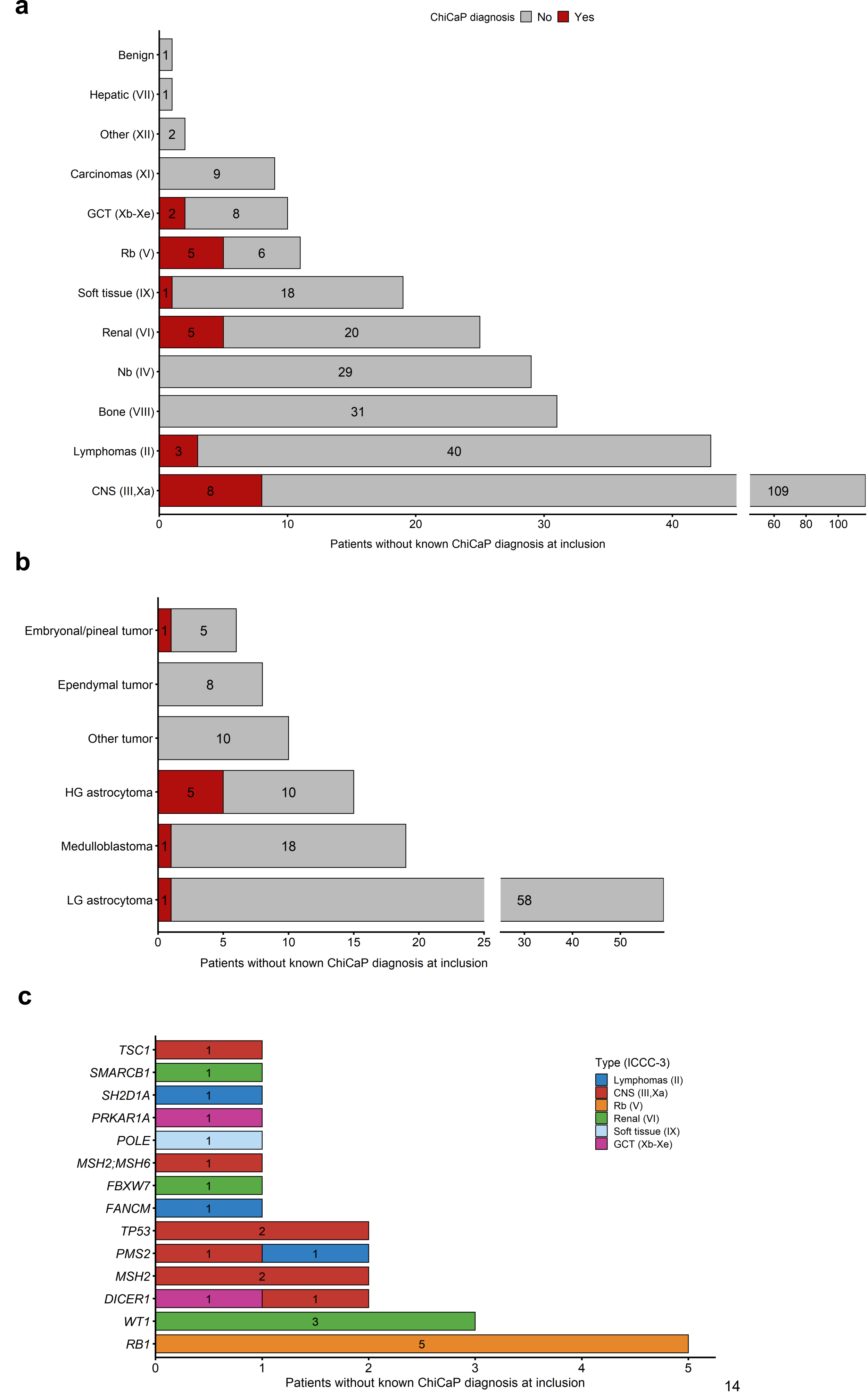

Supplementary Figure 2. Tesi, Lagerstedt Robinson, Abel, et al.

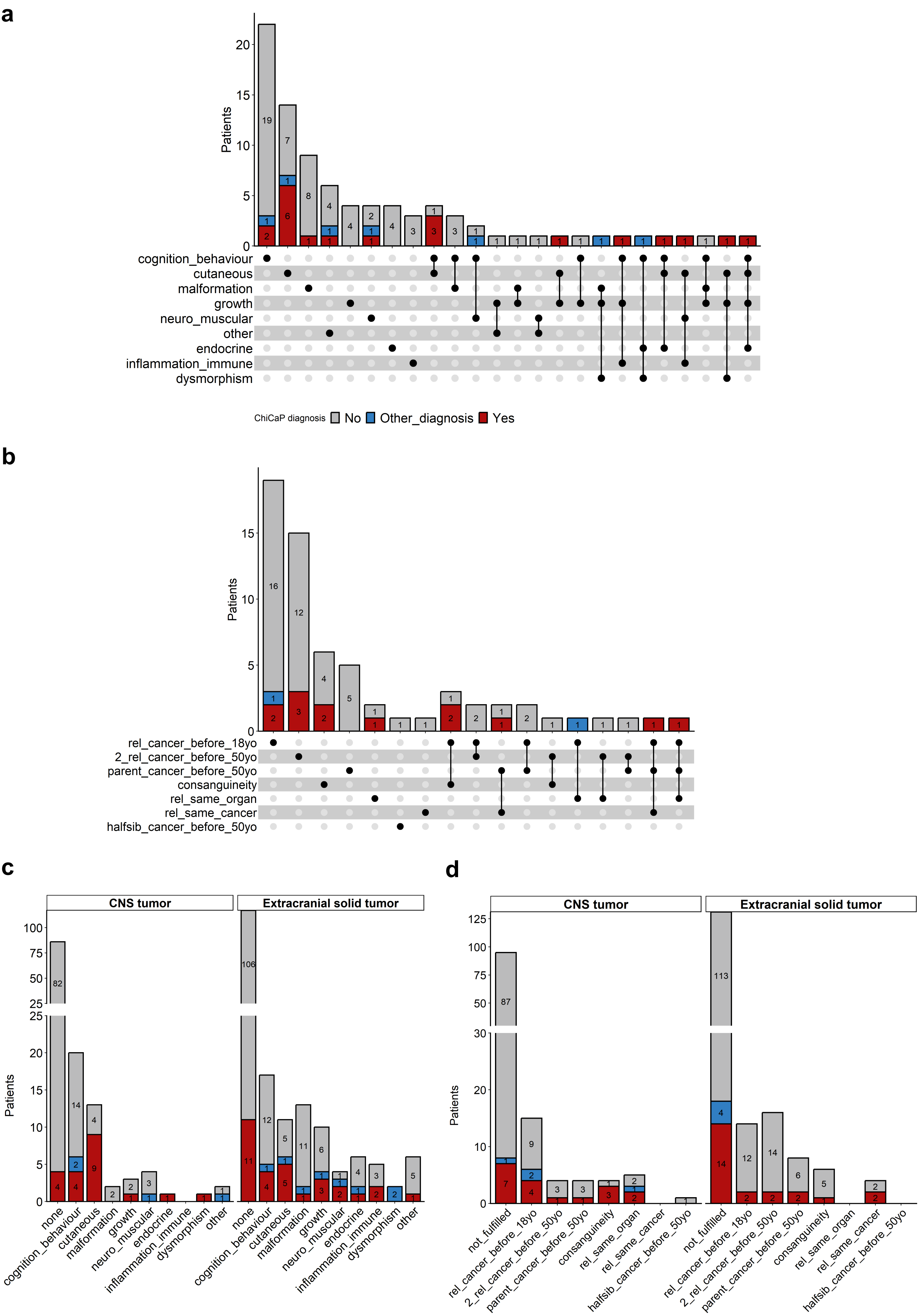

Supplementary Figure 3. Tesi, Lagerstedt Robinson, Abel, et al.

Supplementary table 1. Gene list

HGNC ID	HGNC symbol	Chromosome/scaffold name	Gene start (bp)	Gene end (bp)	Karyotype band	Actionable
HGNC:23336	*A2ML1*	12	8822621	8887001	p13.31	
HGNC:42	*ABCB11*	2	168915498	169031324	q31.1	
HGNC:25070	*ACD*	16	67657512	67660810	q22.1	
HGNC:358	*AIP*	11	67468174	67491154	q13.2	
HGNC:427	*ALK*	2	29192774	29921586	p23.1	
HGNC:583	*APC*	5	112707498	112846239	q22.2	Yes
HGNC:795	*ATM*	11	108223044	108369102	q22.3	Yes
HGNC:950	*BAP1*	3	52401008	52410008	p21.1	
HGNC:1058	*BLM*	15	90717346	90816166	q26.1	Yes
HGNC:1076	*BMPR1A*	10	86756601	86932825	q23.2	Yes
HGNC:1097	*BRAF*	7	140719327	140924929	q34	
HGNC:1100	*BRCA1*	17	43044295	43170245	q21.31	Yes
HGNC:1101	*BRCA2*	13	32315086	32400268	q13.1	Yes
HGNC:20473	*BRIP1*	17	61679139	61863559	q23.2	Yes
HGNC:1149	*BUB1B*	15	40161023	40221123	q15.1	
HGNC:1541	*CBL*	11	119206298	119313926	q23.3	
HGNC:11922	*CD27*	12	6444955	6451718	p13.31	
HGNC:11937	*CD70*	19	6583183	6604103	p13.3	
HGNC:16783	*CDC73*	1	193121983	193254815	q31.2	
HGNC:1786	*CDKN1C*	11	2883213	2885775	p15.4	Yes
HGNC:1833	*CEBPA*	19	33299934	33302534	q13.11	
HGNC:30794	*CEP57*	11	95789965	95832693	q21	
HGNC:2348	*CREBBP*	16	3725054	3880713	p13.3	
HGNC:26169	*CTC1*	17	8224815	8248058	p13.1	
HGNC:2505	*CTLA4*	2	203853888	203873965	q33.2	
HGNC:16850	*CTR9*	11	10751246	10801625	p15.4	
HGNC:2718	*DDB2*	11	47214465	47239217	p11.2	
HGNC:17098	*DICER1*	14	95086228	95158010	q32.13	Yes
HGNC:28648	*DIS3L2*	2	231961245	232344350	q37.1	
HGNC:2890	*DKC1*	X	154762742	154777689	q28	
HGNC:2911	*DLST*	14	74881891	74903743	q24.3	
HGNC:1232	*EGLN1*	1	231363751	231422287	q42.2	
HGNC:14660	*EGLN2*	19	40798996	40808434	q13.2	
HGNC:5959	*ELP1*	9	108866898	108934328	q31.3	
HGNC:3374	*EPAS1*	2	46293667	46386697	p21	
HGNC:11529	*EPCAM*	2	47345158	47387601	p21	
HGNC:3433	*ERCC1*	19	45407334	45478828	q13.32	
HGNC:3434	*ERCC2*	19	45349837	45370918	q13.32	
HGNC:3435	*ERCC3*	2	127257290	127294166	q14.3	
HGNC:3436	*ERCC4*	16	13920138	13952348	p13.12	Yes
HGNC:3437	*ERCC5*	13	102845831	102875995	q33.1	
HGNC:3438	*ERCC6*	10	49454168	49539538	q11.23	
HGNC:3439	*ERCC8*	5	60866454	60945073	q12.1	
HGNC:3495	*ETV6*	12	11649674	11895377	p13.2	
HGNC:3527	*EZH2*	7	148807257	148884321	q36.1	
HGNC:26171	*FAAP100*	17	81539885	81553961	q25.3	Yes
HGNC:3582	*FANCA*	16	89726683	89816977	q24.3	Yes
HGNC:3583	*FANCB*	X	14690388	14873255	p22.2	Yes
HGNC:3584	*FANCC*	9	95099054	95426796	q22.32	Yes
HGNC:3585	*FANCD2*	3	10026370	10101932	p25.3	Yes
HGNC:3586	*FANCE*	6	35452338	35467104	p21.31	Yes
HGNC:3587	*FANCF*	11	22622533	22625823	p14.3	Yes
HGNC:3588	*FANCG*	9	35073835	35080004	p13.3	Yes
HGNC:25568	*FANCI*	15	89243945	89317261	q26.1	Yes
HGNC:20748	*FANCL*	2	58159243	58241410	p16.1	Yes
HGNC:23168	*FANCM*	14	45135930	45200890	q21.2	Yes
HGNC:11920	*FAS*	10	88953813	89029605	q23.31	
HGNC:16712	*FBXW7*	4	152320544	152536092	q31.3	
HGNC:3700	*FH*	1	241497511	241519799	q43	
HGNC:4170	*GATA1*	X	48786540	48794311	p11.23	
HGNC:4171	*GATA2*	3	128479427	128493201	q21.3	

HGNC ID	HGNC symbol	Chromosome/scaffold name	Gene start (bp)	Gene end (bp)	Karyotype band	Actionable
HGNC:4451	*GPC3*	X	133535745	133987100	q26.2	
HGNC:23694	*GPR161*	1	168079542	168137667	q24.2	
HGNC:18437	*HAVCR2*	5	157085422	157142869	q33.3	
HGNC:5173	*HRAS*	11	532242	537321	p15.5	
HGNC:5391	*IDUA*	4	986997	1004564	p16.3	
HGNC:13176	*IKZF1*	7	50304068	50405101	p12.2	
HGNC:6171	*ITK*	5	157142933	157255185	q33.3	
HGNC:16636	*KIF1B*	1	10210570	10381603	p36.22	
HGNC:7133	*KMT2D*	12	49018975	49060794	q13.12	
HGNC:6407	*KRAS*	12	25205246	25250936	p12.1	
HGNC:20499	*L2HGDH*	14	50237563	50312229	q21.3	
HGNC:6601	*LIG4*	13	108207439	108218368	q33.3	
HGNC:6742	*LZTR1*	22	20982269	20999032	q11.21	
HGNC:6764	*MAD2L2*	1	11658918	11691811	p36.22	Yes
HGNC:6840	*MAP2K1*	15	66386837	66491656	q22.31	
HGNC:6842	*MAP2K2*	19	4090321	4124122	p13.3	
HGNC:6871	*MAPK1*	22	21759657	21867680	q11.22	
HGNC:6913	*MAX*	14	65006174	65102695	q23.3	Yes
HGNC:6971	*MDH2*	7	76048051	76067508	q11.23	
HGNC:7010	*MEN1*	11	64803510	64811294	q13.1	Yes
HGNC:7127	*MLH1*	3	36993350	37050846	p22.2	Yes
HGNC:7227	*MRAS*	3	138347648	138405534	q22.3	
HGNC:7325	*MSH2*	2	47403067	47663146	p21	Yes
HGNC:7329	*MSH6*	2	47695530	47810063	p16.3	Yes
HGNC:25126	*NAF1*	4	163110073	163166890	q32.2	
HGNC:7652	*NBN*	8	89924515	90003228	q21.3	Yes
HGNC:7765	*NF1*	17	31094927	31382116	q11.2	Yes
HGNC:7773	*NF2*	22	29603556	29698598	q12.2	Yes
HGNC:14377	*NHP2*	5	178149463	178153894	q35.3	
HGNC:14378	*NOP10*	15	34339159	34343180	q14	
HGNC:7989	*NRAS*	1	114704469	114716771	p13.2	
HGNC:14234	*NSD1*	5	177131830	177300213	q35.3	
HGNC:26144	*PALB2*	16	23603160	23641321	p12.2	Yes
HGNC:8609	*PARN*	16	14435700	14632728	p13.12	
HGNC:8619	*PAX5*	9	36833269	37034268	p13.2	
HGNC:8803	*PDGFRA*	4	54229280	54298245	q12	
HGNC:9143	*PHOX2B*	4	41744082	41748725	p13	
HGNC:8975	*PIK3CA*	3	179148114	179240093	q26.32	
HGNC:9122	*PMS2*	7	5970925	6009130	p22.1	Yes
HGNC:9177	*POLE*	12	132623753	132687376	q24.33	
HGNC:9181	*POLH*	6	43576185	43620523	p21.1	
HGNC:17284	*POT1*	7	124822386	124929983	q31.33	
HGNC:9282	*PPP1CB*	2	28751640	28802940	p23.2	
HGNC:9388	*PRKAR1A*	17	68511780	68551319	q24.2	
HGNC:9585	*PTCH1*	9	95442980	95517057	q22.32	Yes
HGNC:9588	*PTEN*	10	87862563	87971930	q23.31	
HGNC:9644	*PTPN11*	12	112418351	112509918	q24.13	
HGNC:9817	*RAD51*	15	40694774	40732340	q15.1	Yes
HGNC:9820	*RAD51C*	17	58692573	58735611	q22	Yes
HGNC:9829	*RAF1*	3	12582101	12664201	p25.2	
HGNC:9872	*RASA2*	3	141487027	141615344	q23	
HGNC:9884	*RB1*	13	48303744	48599436	q14.2	Yes
HGNC:9949	*RECQL4*	8	144511288	144517845	q24.3	
HGNC:9966	*REST*	4	56907876	56966808	q12	
HGNC:9967	*RET*	10	43077064	43130351	q11.21	Yes
HGNC:25539	*RFWD3*	16	74621399	74666877	q23.1	Yes
HGNC:10023	*RIT1*	1	155897808	155911404	q22	
HGNC:10031	*RMRP*	9	35657754	35658017	p13.3	
HGNC:10301	*RPL11*	1	23691742	23696835	p36.11	
HGNC:10306	*RPL15*	3	23916591	23924374	p24.2	
HGNC:10310	*RPL18*	19	48615328	48619184	q13.33	
HGNC:10316	*RPL23*	17	38847860	38853764	q12	

HGNC ID	HGNC symbol	Chromosome/scaffold name	Gene start (bp)	Gene end (bp)	Karyotype band	Actionable
HGNC:10327	*RPL26*	17	8377516	8383213	p13.1	
HGNC:10328	*RPL27*	17	42998273	43002959	q21.31	
HGNC:10334	*RPL31*	2	101002229	101024032	q11.2	
HGNC:10344	*RPL35*	9	124857880	124861981	q33.3	
HGNC:10345	*RPL35A*	3	197950190	197956610	q29	
HGNC:13631	*RPL36*	19	5674947	5691875	p13.3	
HGNC:10360	*RPL5*	1	92832013	92841924	p22.1	
HGNC:10369	*RPL9*	4	39452587	39458931	p14	
HGNC:10383	*RPS10*	6	34417454	34426069	p21.31	
HGNC:10388	*RPS15*	19	1438358	1440495	p13.3	
HGNC:10389	*RPS15A*	16	18781295	18790383	p12.3	
HGNC:10397	*RPS17*	15	82536750	82540459	q25.2	
HGNC:10402	*RPS19*	19	41860255	41872925	q13.2	
HGNC:10411	*RPS24*	10	78033760	78056813	q22.3	
HGNC:10414	*RPS26*	12	56041351	56044697	q13.2	
HGNC:10416	*RPS27*	1	153990762	153992155	q21.3	
HGNC:10417	*RPS27A*	2	55231903	55235853	p16.1	
HGNC:10418	*RPS28*	19	8321158	8323340	p13.2	
HGNC:10419	*RPS29*	14	49570984	49599164	q21.3	
HGNC:10426	*RPS5*	19	58386400	58394806	q13.43	
HGNC:10440	*RPS7*	2	3575260	3580920	p25.3	
HGNC:10447	*RRAS*	19	49635292	49640143	q13.33	
HGNC:17271	*RRAS2*	11	14277922	14364506	p15.2	
HGNC:15888	*RTEL1*	20	63657810	63696253	q13.33	
HGNC:10471	*RUNX1*	21	34787801	36004667	q22.12	
HGNC:1348	*SAMD9*	7	93099513	93118023	q21.2	
HGNC:1349	*SAMD9L*	7	93130056	93148385	q21.2	
HGNC:19440	*SBDS*	7	66987680	66995693	q11.21	
HGNC:10680	*SDHA*	5	218303	257082	p15.33	Yes
HGNC:26034	*SDHAF2*	11	61430042	61446733	q12.2	Yes
HGNC:10681	*SDHB*	1	17018722	17054032	p36.13	Yes
HGNC:10682	*SDHC*	1	161314381	161363206	q23.3	Yes
HGNC:10683	*SDHD*	11	112086824	112120016	q23.1	Yes
HGNC:15573	*SETBP1*	18	44680173	45068510	q12.3	
HGNC:10820	*SH2D1A*	X	124227868	124373197	q25	
HGNC:15454	*SHOC2*	10	110919367	111017307	q25.2	
HGNC:23845	*SLX4*	16	3581181	3611606	p13.3	Yes
HGNC:6770	*SMAD4*	18	51028394	51085045	q21.2	Yes
HGNC:11100	*SMARCA4*	19	10960932	11079426	p13.2	
HGNC:11103	*SMARCB1*	22	23786931	23838009	q11.23	
HGNC:11109	*SMARCE1*	17	40624962	40648654	q21.2	
HGNC:11187	*SOS1*	2	38962206	39124345	p22.1	
HGNC:11188	*SOS2*	14	50117130	50231578	q21.3	
HGNC:11389	*STK11*	19	1177558	1228431	p13.3	Yes
HGNC:26200	*STN1*	10	103856806	103918332	q24.33	
HGNC:16466	*SUFU*	10	102503972	102633535	q24.32	Yes
HGNC:11727	*TERC*	3	169764520	169765060	q26.2	
HGNC:11730	*TERT*	5	1253147	1295068	p15.33	
HGNC:11824	*TINF2*	14	24238286	24242663	q12	
HGNC:26038	*TMEM127*	2	96248514	96265997	q11.2	Yes
HGNC:11998	*TP53*	17	7661779	7687538	p13.1	Yes
HGNC:16384	*TRIM28*	19	58544064	58550722	q13.43	
HGNC:7523	*TRIM37*	17	58982638	59106921	q22	
HGNC:12307	*TRIP13*	5	892884	919357	p15.33	
HGNC:12362	*TSC1*	9	132891348	132946874	q34.13	Yes
HGNC:12363	*TSC2*	16	2047967	2089491	p13.3	Yes
HGNC:25455	*TSR2*	X	54440404	54448032	p11.22	
HGNC:25009	*UBE2T*	1	202331544	202341984	q32.1	Yes
HGNC:12687	*VHL*	3	10141778	10153667	p25.3	Yes
HGNC:12731	*WAS*	X	48676596	48691431	p11.23	
HGNC:25522	*WRAP53*	17	7686071	7703502	p13.1	
HGNC:12791	*WRN*	8	31033788	31176138	p12	

HGNC ID	HGNC symbol	Chromosome/scaffold name	Gene start (bp)	Gene end (bp)	Karyotype band	Actionable
HGNC:12796	*WT1*	11	32387775	32435564	p13	Yes
HGNC:12814	*XPA*	9	97674909	97697340	q22.33	
HGNC:12816	*XPC*	3	14145147	14178621	p25.1	
HGNC:12829	*XRCC2*	7	152644776	152676193	q36.1	Yes

Supplementary table 5. Somatic findings in patients with retinoblastoma without germline *RB1* variants

Study ID	Type of tumor analysis	Somatic *RB1* variants (VAF)	Other somatic copy number changes	Other somatic SNVs (VAF)	Surveillance of siblings recommended (Yes/No)
BC193	WES FFPE	*Allele 1:* p.Arg556Ter (0.817)<br>*Allele 2*: 13q14.2-	1q+, 6p+	-	No
BC196	WES FFPE	*Allele 1*: 13q14.2 deletion<br>*Allele2*: 13q14.2 deletion	-	-	No
BC198	WGS	Allele 1:RB1::TENM4 (0.30)<br>Allele 2: 13q-	1q+, 2p+, 6p+, ter7p-, ter9q+, ter13q+, ter14q-, 16q-, 17q+	-	No
BC201	WES FFPE	Allele 1: p.Arg556Ter (0.23)<br>Allele 2: ?	bad quality, not possible to determine CNVs	CDKN2B:p.Asn41Ser (0.47)	Yes
BC207	WGS	*Allele 1*: 13q14.2 deletion<br>*Allele2*: 13q14.2 deletion	1q+, ter2q-, 6p+, ter16q-	BCOR: c.3052-1G>T (0.76)	No
BC209	WGS	*Allele 1*: p.Glu629Aspfs*23(0.96)<br>*Allele 2*: CN-LOH 13	3q+, 5p+, 6p+, int 8+, 14+, ter17q+	-	No
BC312*	WGS	*Allele 1*: p.Arg358Ter (0.46)<br>*Allele 2*: ?	1q+, 2p+, 6p+, 10p-, 11p+, 16q-	-	Yes
BC313*	Not available	N.A.	N.A	N.A	Yes
BC314*	WGS	*Allele 1*: p.Arg358Ter (0.99)<br>*Allele 2:* CN-LOH 13	Ter1p- 1q+, ter5q-, 6p+, 12p-, 16q-, ter 19q+	-	No
BC315*	WGS	Allele 1: p.Arg455Ter (0.39)<br>Allele 2: c.2490-1G>A (0.46)	-	-	No

*Included after 1st january 2023. N.A., Not applicable. VAF, Variant allele frequency. CN-LOH, Copy-neutral loss of heterozygosity. Term, terminal. Int, interstitial.
